# CF10/LV overcomes acquired resistance to 5-FU/LV in colorectal cancer cells through downregulation of the c-Myc/ABCB5 axis

**DOI:** 10.20517/cdr.2025.76

**Published:** 2025-07-15

**Authors:** Charles Chidi Okechukwu, William H. Gmeiner

**Affiliations:** ^1^Integrative Physiology and Pharmacology Graduate Program, Wake Forest University School of Medicine, Winston-Salem, NC 27157, USA.; ^2^Department of Cancer Biology, Wake Forest University School of Medicine, Winston-Salem, NC 27157, USA.

**Keywords:** Fluoropyrimidine, thymidylate synthase, DNA Topoisomerase 1, Myc, ABCB5, CF10

## Abstract

**Aim:** Acquired resistance to 5-fluorouracil/leucovorin (5-FU/LV) frequently develops during treatment of metastatic colorectal (mCRC), but the causes are incompletely understood. We aim to: (i) identify the causes of 5-FU/LV resistance under physiological folate; and (ii) determine if a polymeric fluoropyrimidine (FP) CF10 remains potent to CRC cells selected for 5-FU/LV resistance.

**Methods:** 5-FU/LV-resistant CRC cells were selected by repeated passaging with increasing 5-FU/LV concentrations, and resistance factors were calculated from dose-response studies. Basal and treatment-induced thymidylate synthase (TS), Myc, and ABCB5 were determined by RT-qPCR and Western blot. TS activity was determined using an *in situ* ^3^H-release assay. DNA topoisomerase 1 cleavage complexes (Top1cc) and DNA double-strand breaks (DSBs) were determined by immunofluorescence.

**Results:** Acquired resistance to 5-FU/LV with physiological folate was associated with a <1.5-fold increase in basal TS levels; however, with either 5-FU/LV or CF10/LV treatment, TS levels were elevated ~5-fold by Western blot but only ~2-fold by RT-qPCR. CF10 remained very potent to CRC cells selected for 5-FU/LV resistance, and CF10 effectively induced TS ternary complex formation and inhibited TS catalytic activity in 5-FU/LV-resistant CRC cells. c-Myc was expressed at ~4-fold higher levels in 5-FU/LV-resistant CRC cells, but Myc was barely detectable with CF10/LV treatment. The Myc-target ABCB5, which is an established factor in resistance to 5-FU and other drugs, was substantially downregulated with CF10/LV but not 5-FU/LV treatment.

**Conclusion:** Acquired 5-FU/LV resistance was associated with FP-induced TS and elevated Myc and ABCB5. There is minimal cross-resistance to CF10 in 5-FU/LV-resistant CRC cells, consistent with its use in treating 5-FU/LV-resistant mCRC.

## INTRODUCTION

**Figure 1:**
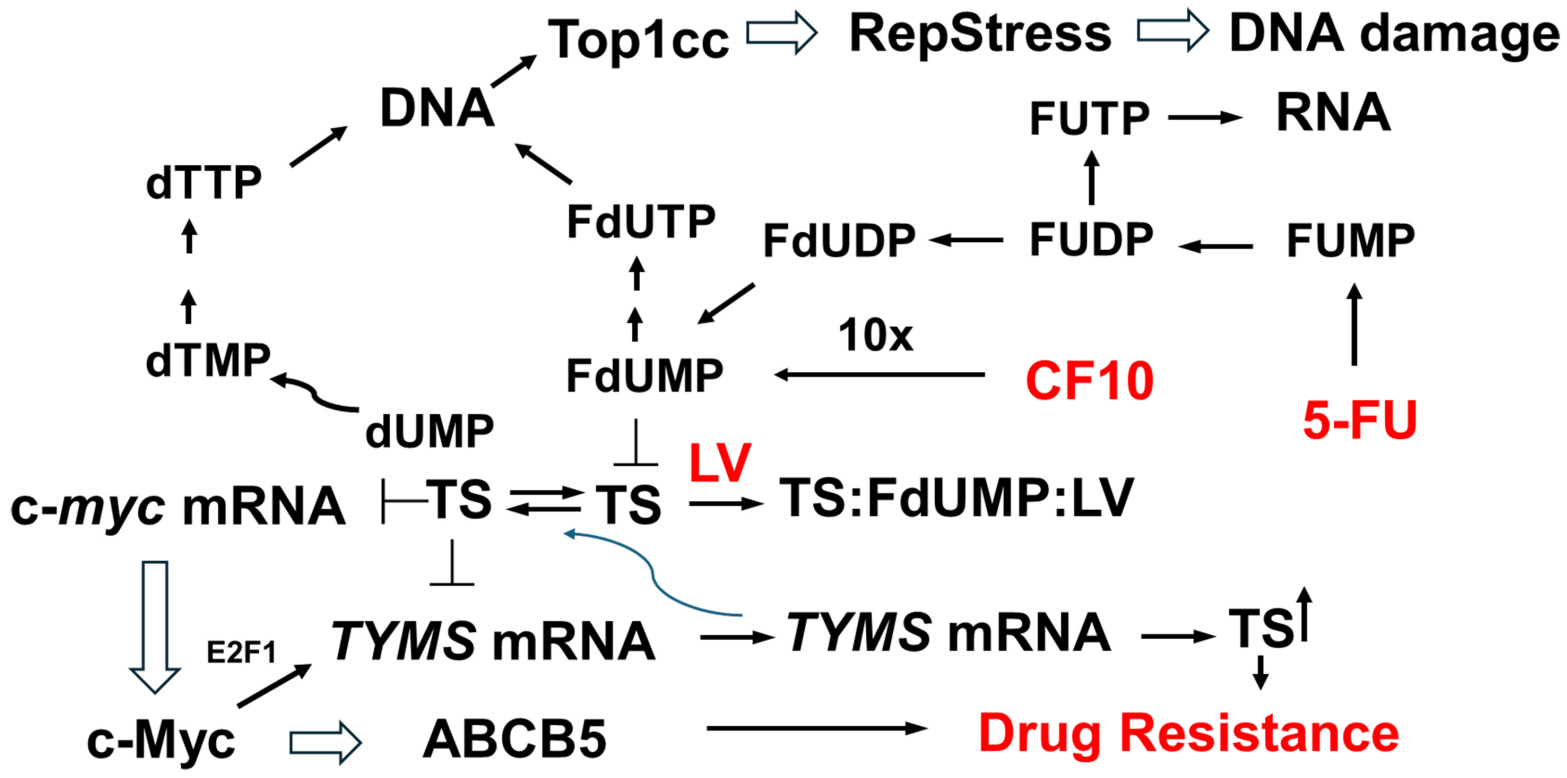
Resistance to 5-FU/LV converges on a lack of efficacy in inhibiting TS due to: (i) elevated TS expression; (ii) inefficient conversion to the TS inhibitory metabolite FdUMP; and/or (iii) activation of the c-Myc/ABCB5 axis. CF10 is efficiently converted to FdUMP, resulting in strong TS inhibition even when the enzyme is expressed at elevated levels. Figure preparation software is Microsoft PowerPoint (RRID: SCR_023631). 5-FU: 5-Fluorouracil; LV: leucovorin; TS: thymidylate synthase.

The fluoropyrimidine (FP) drug 5-fluorouracil (5-FU) is central to the management of metastatic colorectal cancer (mCRC), with 5-FU-based regimens (FOLFOX, FOLFIRI) being administered to most mCRC patients^[1]^. While the majority of mCRC patients initially respond to 5-FU-based chemotherapy, acquired resistance develops in nearly all patients^[2]^ [Figure 1]. Thus, while survival for mCRC patients enrolled in clinical trials now exceeds 30 months, during which many patients receive three or more lines of FP-based chemotherapy, long-term survival remains rare^[3]^. An increased understanding of the causes of acquired 5-FU resistance thus remains an important objective in cancer research, as does the development of next-generation FP therapeutics to overcome the limitations of current 5-FU-based therapy^[4]^.

The primary molecular target of FP chemotherapy is thymidylate synthase (TS)^[5,6]^. TS is required for *de novo* thymidylate biosynthesis, which promotes rapid cell proliferation in cancer cells. TS enzymatic activity requires the substrate dUMP and a reduced folate cofactor, N^5^,N^10^-methylene tetrahydrofolate (MeTHF), that serves as a carbon donor^[7]^. The FP metabolite FdUMP forms a covalent complex with TS that inhibits enzymatic activity^[8]^; however, 5-FU is inefficiently converted to FdUMP, with < 5% of the administered 5-FU converted to FdUMP^[9]^. Irreversible TS inhibition also requires formation of a ternary complex that also includes a reduced folate cofactor^[10]^. Humans have relatively low folate levels compared to rodents^[11]^, and folate levels are particularly low in cancer patients^[12,13]^. For this reason, 5-FU is nearly always administered with LV (N^5^-formyl tetrahydrofolate) or newer reduced folate analogs that do not require metabolism^[14]^ for cancer treatment to promote TS inhibition despite 5-FU’s inefficient conversion to FdUMP^[15]^.

Multiple studies have investigated the mechanistic basis for 5-FU resistance in CRC^[2,16-18]^. A common theme among the majority of studies is that 5-FU resistance generally results from decreased TS inhibition that is due to either: (i) increased TS levels^[19]^; (ii) decreased conversion of 5-FU to FdUMP^[20]^; or (iii) increased breakdown of FdUMP or its cellular efflux [Figure 1]. To overcome resistance to 5-FU, we developed CF10^[21-24]^, a second-generation FP polymer that directly releases FdUMP. CF10 is much more potent than 5-FU against CRC cells (>300-fold on average in the NCI60 cell line screen)^[21]^, and this increased potency corresponds to stronger TS inhibition by CF10 at correspondingly lower doses relative to 5-FU. In previous studies, we have shown that FP polymers such as CF10 are highly effective in CRC cells selected for acquired resistance to 5-FU due to elevated TS expression^[25]^.

In addition to its enzymatic function, TS is an RNA-binding protein that autoregulates its own expression and the expression of other proteins, notably c-Myc and p53, through mRNA binding and translational repression^[26]^. The function of TS as an RNA-binding protein is modulated by the formation of the FdUMP/MeTHF/TS ternary complex, which relieves autoregulation to enable increased TS expression under conditions of TS inhibition^[27]^. The binding affinity of TS for its own mRNA and for c-*myc* is similar^[28]^. Hence, strong TS inhibition that reduces binding of TS mRNA could decrease c-Myc levels due to increased binding of c-*myc* mRNA by TS, inhibiting its translation. Decreased Myc levels in turn alter its translational program that is fundamental to determining the balance between proliferation and cell death^[29]^. Among the Myc targets that have an established role in mediating resistance to 5-FU^[30]^ and other drugs is ABCB5^[31]^, which is involved in promoting a cancer stem cell phenotype^[32]^.

The purpose of this study is to characterize the role of TS elevation in CRC cells selected for acquired resistance to 5-FU/leucovorin (LV) under conditions of human-like folate levels and to determine if CF10 remains effective in these cells. Although multiple previous studies have investigated acquired 5-FU resistance^[2,33-35]^ and shown increased TS and *TYMS* levels, this is the first study to adapt cells to culture medium that includes human-like folate levels^[36]^ and to select for resistance to 5-FU/LV under conditions similar to those encountered clinically. We find that in multiple CRC cell lines, there is minimal constitutive change in TS at either the protein or mRNA level, but that 5-FU/LV treatment results in elevated TS levels that contribute to 5-FU/LV resistance. Given the interplay between TS and Myc regulation^[26]^, we also investigate perturbation in Myc^[37]^ and its direct transcriptional target ABCB5. We find that CF10 remains effective in CRC cells selected for 5-FU/LV resistance despite causing similar induction in TS, and this retained potency is associated with downregulation of c-Myc and ABCB5 at both the mRNA and protein levels.

## METHODS

### Cell lines and drugs

All parental colorectal cancer (CRC) cells [HCT116 (RRID:CVCL_0291), HCT15 (RRID:CVCL_0292), LS174T (RRID:CVCL_1384), and MC38 (RRID:CVCL_B288)] and 5FU-LV-resistant CRC cells (HCT116^R^, HCT15^R^, LS174T^R^, MC-38^R^) were cultured using folate-restricted (FR) media (folic acid-free RPMI 1640 supplemented with 40 nM of 5-methyltetrahydrofolate and 0.1 nM of sodium L-ascorbate)^[36]^, confirmed by short tandem repeat analysis, and repeatedly tested negative for Mycoplasma. The 5FU/LV-resistant CRC cells were generated by repeated passaging over 40 weeks in FR media supplemented with 5FU (0.1 to 10 µM) and LV (10 µM). Clinical-grade 5-FU (50 mg/mL) was purchased from the Baptist Hospital clinical pharmacy, and its concentrations were calculated based on the dilution of the established stock concentration. CF10 was obtained from ST Pharma, confirmed by high-resolution mass spectrometry, and dissolved in 0.9% sterile saline. Its concentrations were matched to deliver equivalent nucleoside content to an established dose of 5-FU based on UV absorbance at 260 nm.

### Cell viability and resistance assay

CRC^P^ and CRC^R^ cells were seeded into white, flat-bottom 96-well plates and allowed to adhere and proliferate for 48 h until reaching about 25% confluency. To test for 5-FU/LV resistance, 2,500 cells per well, both the CRC Parental (CRC^P^) and 5-FU/LV-resistant (CRC^R^) cells, were seeded in 96-well plates and allowed to attach for 24 h. After 24 h, the cells were pre-treated for 2 h with LV before adding 5-FU and then incubated for 96 h at 37 ^o^C in 5% CO_2_. Cell viability was assessed using Aqueous-one (Promega) following the manufacturer’s instructions. Resistance factors were calculated based on the ratio of IC50 values for CRC^P^ and CRC^R^ cells. All experiments were performed in triplicate.

### Clonogenic assay

A modified clonogenic assay assessed the potency of CF10 and 5-FU in CRC^P^ and CRC^R^ cells. The CRC cells were plated in 24-well plates in FR media, and after 24 h, they were treated with the indicated concentration of CF10 or 5-FU for 72 h. The media were replaced after drug treatment, while the cells were allowed to grow for 168 h. Cell proliferation was evaluated using the Aqueous One (Promega) reagent, following the manufacturer’s instructions, after the cell solution was transferred to 96-well plates. Apoptosis was evaluated using Caspase 3/7-Glo reagent (Promega) following the manufacturer’s instructions.

### Western blotting

Proteins were isolated, and their differential expressions were analyzed by Western blot, as described previously^[23]^. Briefly, both CRC^P^ and CRC^R^ cells treated with LV, 5FU, 5FU + LV, CF10, or CF10 + LV were lysed using RIPA buffer (50 mM Tris HCl at pH 7.4, 150 mM NaCl, 1% Triton X-100 or NP-40, 0.5% sodium deoxycholate, 0.1% SDS, 1 mM EDTA, and 10 mM NaF freshly supplemented with protease and phosphatase inhibitors). The protein concentrations were quantified using a Bradford assay (Bio-Rad), and the samples were normalized for equal loading. All samples were then heated to 95 °C for 10 min in the presence of 6× Laemmli buffer (Boston Bio-Products; Milford, MA, USA). The proteins were resolved using SDS-PAGE, and their expression was analyzed following immunoblotting using specific antibodies. The following antibodies were used in this study: TS (Cell Signaling Technology Cat# 3766, RRID: AB_2210584), Myc (Cell Signaling Technology Cat# 18583, RRID:AB_2895543), ABCB5 (Thermo Fisher Scientific Cat# PA5-114801, RRID:AB_2899437), and β-actin (Santa Cruz Biotechnology Cat# sc-47778, RRID: AB_626632).

### Immunoprecipitation assay

CRC^R^ treated with LV or 5FU + LV and non-treated cells were exposed to 100 nM control siRNA (Cell Signaling Technology Cat# 6568) and c-Myc siRNA (Cell Signaling Technology Cat# 6341) for 48 h as recommended by the vendor. The cells were lysed using RIPA buffer (50 mM Tris HCl at pH 7.4, 150 mM NaCl, 1% Triton X-100 or NP-40, 0.5% sodium deoxycholate, 0.1% SDS, 1 mM EDTA, and 10 mM NaF freshly supplemented with protease and phosphatase inhibitors). Immunoprecipitation [Thermo Scientific Pierce Co-Immunoprecipitation (Co-IP) Kit Cat# 26149] was performed using the cell lysates and ABCB5 (Thermo Fisher Scientific Cat# PA5-114801, RRID:AB_2899437) antibody. The enriched eluted lysates were resolved using SDS-PAGE and probed for ABCB5 protein expression (Thermo Fisher Scientific Cat# PA5-114801, RRID:AB_2899437).

#### Immunofluorescence Assay

Immunofluorescence detection of pH2AX (Cell Signaling Technology Cat# 2577, RRID:AB_2118010) was performed in drug-treated CRC^P^ and CRC^R^ cells that were fixed and permeabilized using 100% cold methanol and then processed as previously described^[38]^. Top1cc were detected using a primary antibody specific to the cleavage complex (gift of Dr. S. Kaufmann) using procedures like those previously described^[38]^. Top1cc foci were captured using Olympus Confocal Laser Scanning Microscope Fluoview FV3000 (RRID:SCR_017015) and quantification of Top1cc foci was done via ImageJ by counting at least 100 cells that were declared positive if they had > 50 foci per nucleus.

#### RNA isolation and real-time PCR

Total RNA was isolated from CRC^P^ and CRC^R^ cells treated with LV, 5-FU, 5-FU + LV, CF10, or CF10 + LV using the RNeasy mini kit QIAGEN (RRID:SCR_008539). The Thermo Scientific NanoDrop One/OneC Microvolume UV Vis Spectrophotometer (RRID:SCR_023005) was used to determine the quality and quantity of RNA samples. cDNA synthesis was performed using the iScript^TM^ cDNA Synthesis Kit (Bio-Rad #:1708891). Real-time PCR was performed using the Bio-Rad CFX96 Real-Time PCR Detection System (RRID:SCR_018064) and the iTaq SYBR Green supermix (Bio-Rad #: 1725121). The primers for qRT-PCR were all purchased from OriGene Technologies Inc.

TS (human): Forward: 5′-GGTGTTTTGGAGGAGTTGCTGTG-3′, Reverse: 5′-GGAGAATCCCAGGCTGTCCAAA-3′; Myc (human): Forward: 5′-CCTGGTGCTCCATGAGGAGAC-3′, Reverse: 5′-CAGACTCTGACCTTTTGCCAGG-3′; ABCB5 (human): Forward: 5′-TTTGCCTATGCGGCAGGGTTTC-3′, Reverse: 5′-CAAAACGAGCGTTTCTCCGATGG-3′; β-actin (human): Forward: 5′- CACCATTGGCAATGAGCGGTTC-3′, Reverse: 5′-AGGTCTTTGCGGATGTCCACGT-3′.

β-actin were used as an internal standard to normalize the relative expressions of the mRNAs. The RT-PCR reaction settings were Stage 1: Activation: 50 °C for 2 min; Stage 2: pre-soak:95 °C for 10 min; Stage 3: Denaturation: 95 °C for 15 s, Annealing: 60 °C for 1 min for 40 cycles; Stage 4: Melting curve: 95 °C for 15 s, 60 °C for 15 s, 95 °C for 15 s. The relative expressions were analyzed by the 2^-ΔΔCt^ method and experiments were performed in triplicate.

#### TS activity assay

A TS *in situ* activity assay was performed as previously described^[39]^ (PMID: 10554030) to quantitatively assess intracellular TS inhibition following drug treatment. Approximately 0.5 × 10^6^ HCT-116 (RRID:CVCL_0291) and HCT116R cells were plated in each well of 6-well plates and allowed to attach for 24 h, then treated with either LV, 5-FU, 5-FU + LV, CF10, or CF10 + LV for 48 h. For the last 2 h, [5-^3^H]-2’-deoxyuridine (Moravek; 0.16 Ci/mmol; final concentration 2.5 µM) was added. After incubation, 150 µL of the culture media from each well was added to an ice-cold suspension containing 750 µL of charcoal with 0.5% T-70 dextran and 2.5% BSA, along with 150 µL of 35% trichloroacetic acid. The samples were centrifuged, and a portion of the supernatant was counted by liquid scintillation counting, and enzyme activities were expressed as a percentage relative to the drug-free control.

#### Statistical analysis

GraphPad Prism 10.2.0 GraphPad Prism (RRID:SCR_002798) was used for all statistical analyses. All experiments were conducted in triplicate, and data are presented as mean ± SEM. One-way ANOVA tests with recommended Tukey corrections were conducted, with *P* < 0.05 indicating significance.

## RESULTS

### Acquired resistance to 5-FU/LV occurs through treatment-induced TS elevation

**Figure 2:**
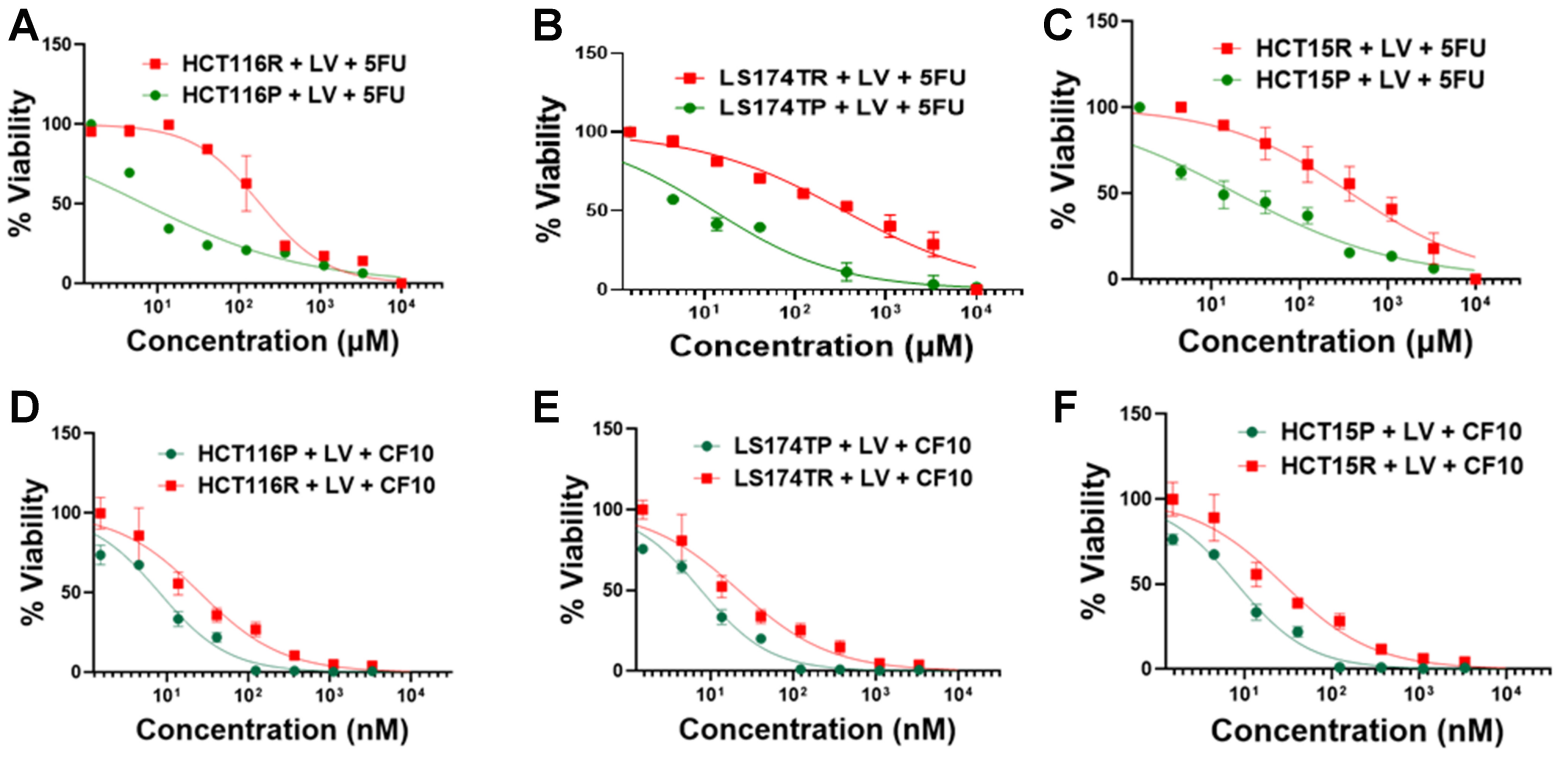
Dose response of parental (green) and 5-FU/LV-resistant (red) CRC cells to 5-FU/LV in (A) HCT116 (*P* = 0.0022), (B) LS174T (*P* = 0.0028), and (C) HCT15 (*P* < 0.0001) cells. Dose response to CF10/LV is shown in (D) HCT116 (*P* = 0.0337), (E) LS174T (*P* = 0.0009), and (F) HCT15 (*P* = 0.0112) parental (green) and 5-FU/LV-resistant (red) cells. IC50 values and resistance factors for each CRC cell line are summarized in Table 1. Figure preparation software is GraphPad Prism (RRID: SCR_002798). 5-FU: 5-Fluorouracil; LV: leucovorin; CRC: colorectal cancer.

In multiple previous studies, 5-FU-resistant CRC cells were generated through repeated passaging in media containing increasing concentrations of 5-FU^[2,33-35]^. However, in the present studies, we repeatedly passaged human CRC cells (HCT116, LS174T, HCT15) in the presence of 5FU/LV, as 5-FU is nearly always used with LV in combination chemotherapy regimens for CRC. We also performed the selection by repeated passaging in media with restricted folate levels to better simulate human physiology. After selection by repeated passaging of parental cells (e.g. HCT116^P^) over > 4 months in 5-FU/LV, we tested cells for the extent of resistance and found HCT116 5FU/LV-resistant (HCT116^R^) cells were 27.3-fold less sensitive to 5-FU/LV relative to the corresponding parental cells [Figure 2A and Table 1]. Resistant LS174T and HCT15 cells showed similar resistance factors relative to the corresponding parental cells (27.1- and 19.9-fold) [Figure 2B and C, Table 1]. Importantly, all CRC cell lines selected for resistance to 5-FU/LV remained highly sensitive to CF10 and HCT116^R^ cells were only 3.14-fold less sensitive to CF10 than HCT116^P^ cells [Figure 2D], with other CRC cell lines showing similar moderate changes in sensitivity [Figure 2E and F, Table 1].

**Table 1:** Summary of IC50 values for 5-FU/LV (μM) and CF10/LV (nM) and resistance factors

**CRC cell line**	**(LV + 5FU) Treatment IC50 (µM)**	**Resistance factor**	**(LV + CF10) Treatment IC50 (nM)**	**Resistance factor**
HCT116P	6.70	27.3	8.07	3.14
HCT116R	183		25.3	
LS174TP	12.8	27.1	7.73	2.85
LS174TR	347		22.0	
HCT15P	19.2	19.9	8.23	3.40
HCT15R	383		28.0	

5-FU: 5-Fluorouracil; LV: leucovorin; CRC: colorectal cancer.

**Figure 3:**
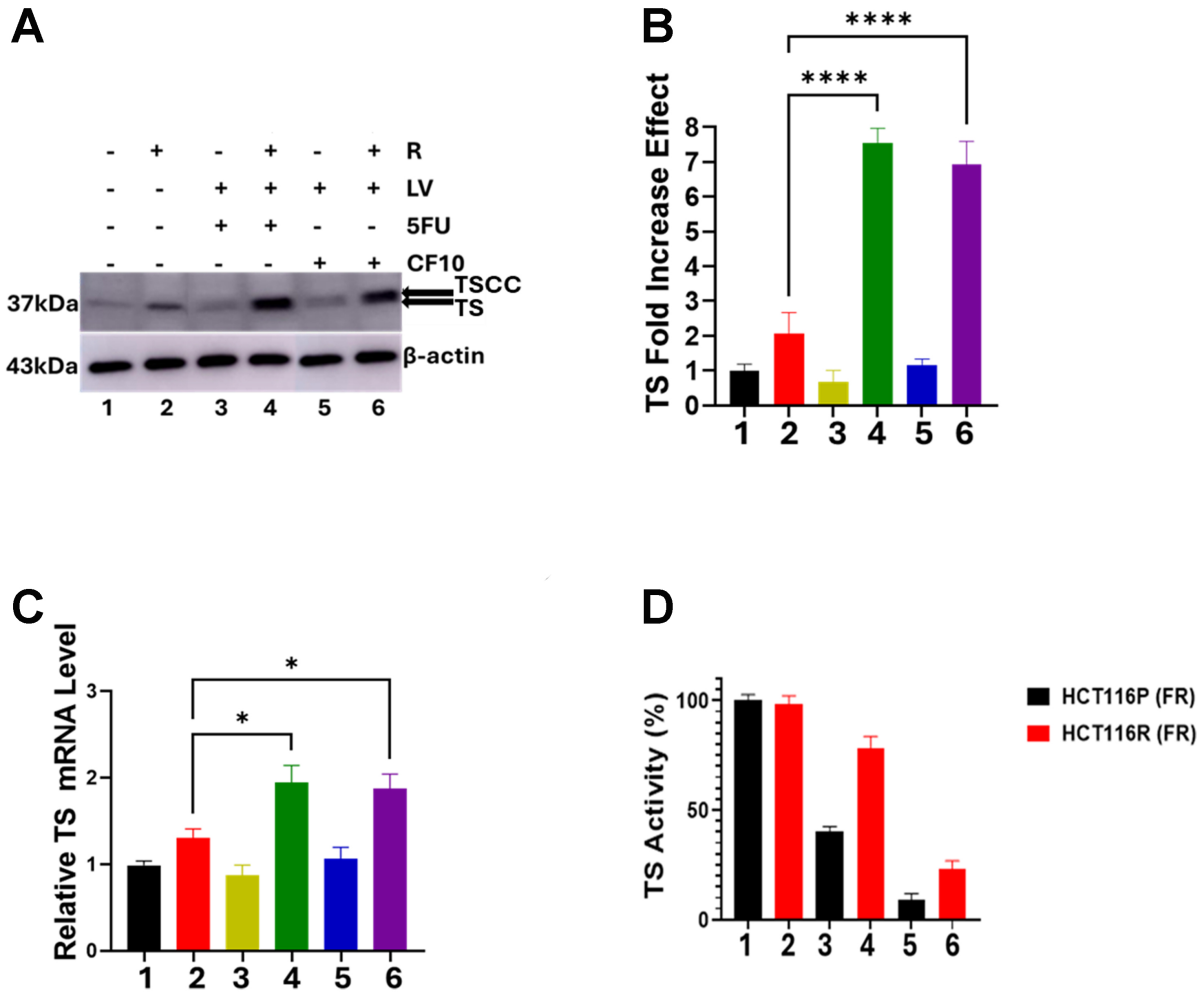
5-FU/LV (10 mM, 1 mM) and CF10/LV (1 mM, 1 mM) treatment for 24 h increase TS at both the protein (A and B) and mRNA (C) levels in HCT116^R^ cells, but only CF10/LV promotes ternary complex formation (TSCC) and efficient inhibition of TS enzymatic activity (D). ^*^P < 0.03; ^****^P < 0.0001. Figure preparation software is GraphPad Prism (RRID: SCR_002798). 5-FU: 5-Fluorouracil; LV: leucovorin; TS: thymidylate synthase; TSCC: TS classic complex.

Since TS is an established target of 5-FU/LV, we performed Western blot and RT-qPCR to determine to what extent resistance in selected cells was associated with increased *TYMS* expression and increased TS protein levels. Quantification of Western blot images revealed that TS was upregulated ~2-fold in HCT116^R^ cells [Figure 3A and B] and similar increases were observed for LS174T [Supplementary Figures 1 and 2] and HCT15 cells [Supplementary Figures 3 and 4]. RT-qPCR revealed a modest increase in *TYMS* mRNA, ~1.2-fold in HCT116^R^ cells [Figure 3C and Supplementary Figure 5] and in LS174T [Supplementary Figures 1 and 2], and HCT15 cells [Supplementary Figures 3 and 4]. We next investigated if TS levels were altered in response to treatment in FU/LV-R cells relative to parental cells. Treatment of HCT116^P^ cells resulted in no significant change in TS mRNA or protein levels under any of the treatment conditions tested. However, TS mRNA levels were increased in HCT116^R^ cells, with a similar ~2-fold elevation observed following both 5-FU/LV and CF10/LV treatments, regardless of the presence or absence of LV co-treatment [Figure 3C]. Similar trends were found for TS protein levels with 5-FU and CF10 treatment [Supplementary Figure 5], but the effects were larger at the protein level (~7-fold) than for *TYMS* mRNA (~2-fold) in HCT116^R^ cells [Figure 3A-C] and the other CRC cell lines selected for 5-FU/LV-resistance [Supplementary Figures 1-4]. Similar increases in TS were not detected with treatment in any of the parental CRC cell lines.

To determine if increased TS levels induced by 5-FU/LV or CF10/LV treatment in FU/LV-R CRC cells were a cause of resistance, we evaluated TS ternary complex formation based on decreased mobility on PAGE gels with Western blot detection [e.g., TS classic complex (TSCC)] [Figure 3A]. We also evaluated TS enzymatic activity using a TS *in-situ* enzyme activity assay (TSIA) based on ^3^H_2_O formation from [5-^3^H]-dUMP [Figure 3D]. TS mobility in untreated and 5-FU/LV-treated cells was similar, indicating that 5-FU/LV treatment (10 mM, 1 mM; 24 h) did not effectively promote TS ternary complex (TSCC) formation in HCT116^R^ cells [Figure 3A]. In contrast, CF10/LV treatment (1 mM, 1 mM; 24 h) reduced TS mobility, consistent with TS ternary complex formation. CF10 was used at a 10-fold lower concentration than 5-FU to deliver equivalent FP levels. TSIA assays further demonstrated that CF10/LV effectively inhibited TS enzymatic activity in both HCT116^P^ and HCT116^R^ cells. Conversely, 5-FU/LV was less effective than CF10/LV at reducing TS enzymatic activity in HCT116^P^ cells and showed no significant inhibition in HCT116^R^ cells [Figure 3D]. Similar trends were observed with single-agent treatments in LS174T and HCT15 cells [Supplementary Figures 1-4] and in HCT116^R^ cells [Supplementary Figure 5]. Collectively, these studies showed that increased TS levels in CRC^R^ cells render 5-FU/LV ineffective at inducing TS ternary complex formation and inhibiting TS enzymatic activity, whereas CF10/LV remains effective in TS inhibition despite higher enzyme levels.

### Myc is elevated in 5-FU/LV-resistant cells and is downregulated by CF10/LV

**Figure 4:**
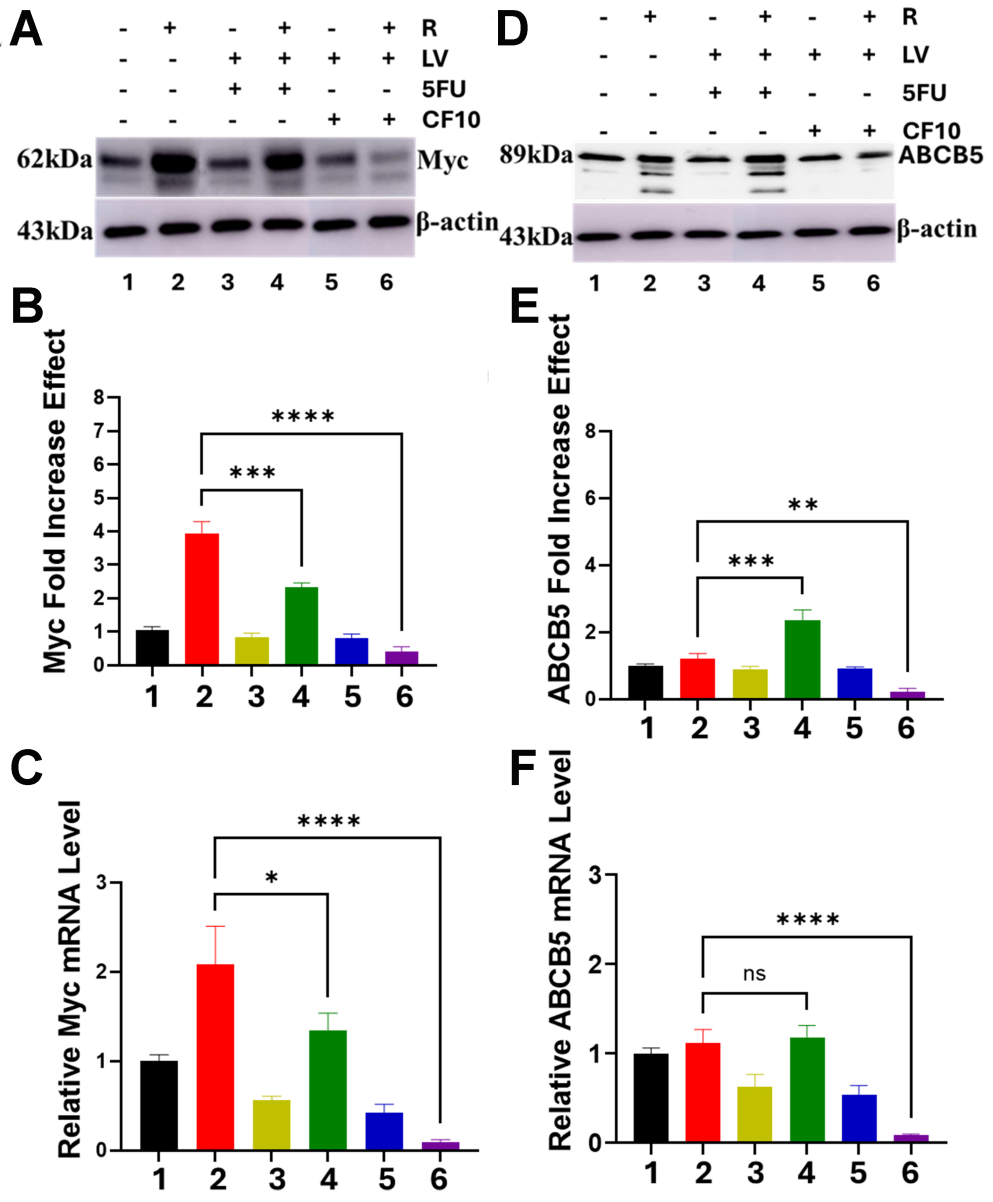
Myc is elevated in 5-FU/LV-resistant HCT116^R^ cells, but CF10/LV decreases levels of Myc and the Myc-target ABCB5 at both the protein and mRNA levels. (A) Western blot for Myc with quantification of the Western blot image by densitometry shown in (B) and quantification of mRNA levels by RT-qPCR shown in (C). (D) Western blot for ABCB5 with quantification of the Western blot image by densitometry shown in (E) and quantification of mRNA levels by RT-qPCR shown in (F). ^*^P < 0.03, ^**^P < 0.002, ^***^P < 0.0002, ^****^P < 0.0001. Figure preparation software is GraphPad Prism (RRID: SCR_002798). 5-FU: 5-Fluorouracil; LV: leucovorin; RT-qPCR: reverse transcription quantitative polymerase chain reaction.

Myc is elevated in multiple cancers, including CRC, driving proliferation and upregulating a transcriptional program that contributes to drug resistance. Myc expression is translationally regulated by TS binding to its cognate mRNA^[28]^; hence, treatments that affect TS levels could also alter Myc, affecting proliferation and drug resistance. The pattern of Myc expression in CRC^R^ cells parallels that for TS at baseline in the absence of treatment, with Myc levels ~4-fold higher at the protein level and ~2-fold higher at the mRNA level in HCT116^R^ cells compared to parental cells [Figure 4A-C, Supplementary Figure 6]. Similar trends were observed in LS174T and HCT15 cells [Supplementary Figures 7-10]. As for TS, treatment of parental cells with either 5-FU/LV or CF10/LV had minimal effect on Myc levels. However, in contrast to the increase in TS levels observed with treatment in HCT116^R^ cells, treatment decreased Myc at both the mRNA and protein levels in HCT116^R^ cells. 5-FU/LV treatment decreased Myc mRNA and protein relative to non-treated control cells; however, Myc levels were still elevated 1.5-fold (mRNA) and 2-fold (protein) in 5-FU/LV-treated HCT116^R^ cells relative to similarly treated HCT116^P^ cells [Figure 4A-C]. The effect of CF10/LV on Myc levels in HCT116^R^ cells was striking, with Myc barely detectable at either the mRNA or protein level in HCT116^R^ cells with CF10/LV treatment [Figure 4A-C]. Similar effects of CF10/LV on the reduction of Myc levels were detected in LS174T^R^ and HCT15^R^ cells [Supplementary Figures 7-10].

The effects of elevated Myc in mediating resistance to 5-FU and other drugs have implicated ABCB5 as an important direct target of Myc that mediates drug resistance^[30]^. To determine if ABCB5 levels were altered in CRC cells selected for 5-FU/LV acquired resistance, we evaluated ABCB5 levels both by Western blot [Figure 4D and E, Supplementary Figure 6] and RT-qPCR [Figure 4F and Supplementary Figure 6]. The principal difference in Western blots for ABCB5 in 5-FU/LV-resistant cells relative to parental cells was the appearance of three additional bands with slightly increased mobility relative to the major band detected at ~89 kDa. ABCB5 undergoes alternative splicing to produce multiple protein products, and the new bands detected in 5-FU/LV-resistant CRC cells are likely due to alternatively spliced products (proteomics analysis is in progress). Treatment with 5-FU/LV resulted in slight intensification of one band [Figure 4D and E]. In contrast, none of the additional bands attributed to ABCB5 alternative splicing were detected in HCT116^R^ cells treated with CF10/LV. Furthermore, CF10/LV treatment reduced ABCB5 mRNA levels to barely detectable levels, similar to what was observed for Myc mRNA in HCT116^R^ cells following CF10/LV treatment.

### Myc siRNA knockdown decreases ABCB5 in CRC^R^ cells

**Figure 5:**
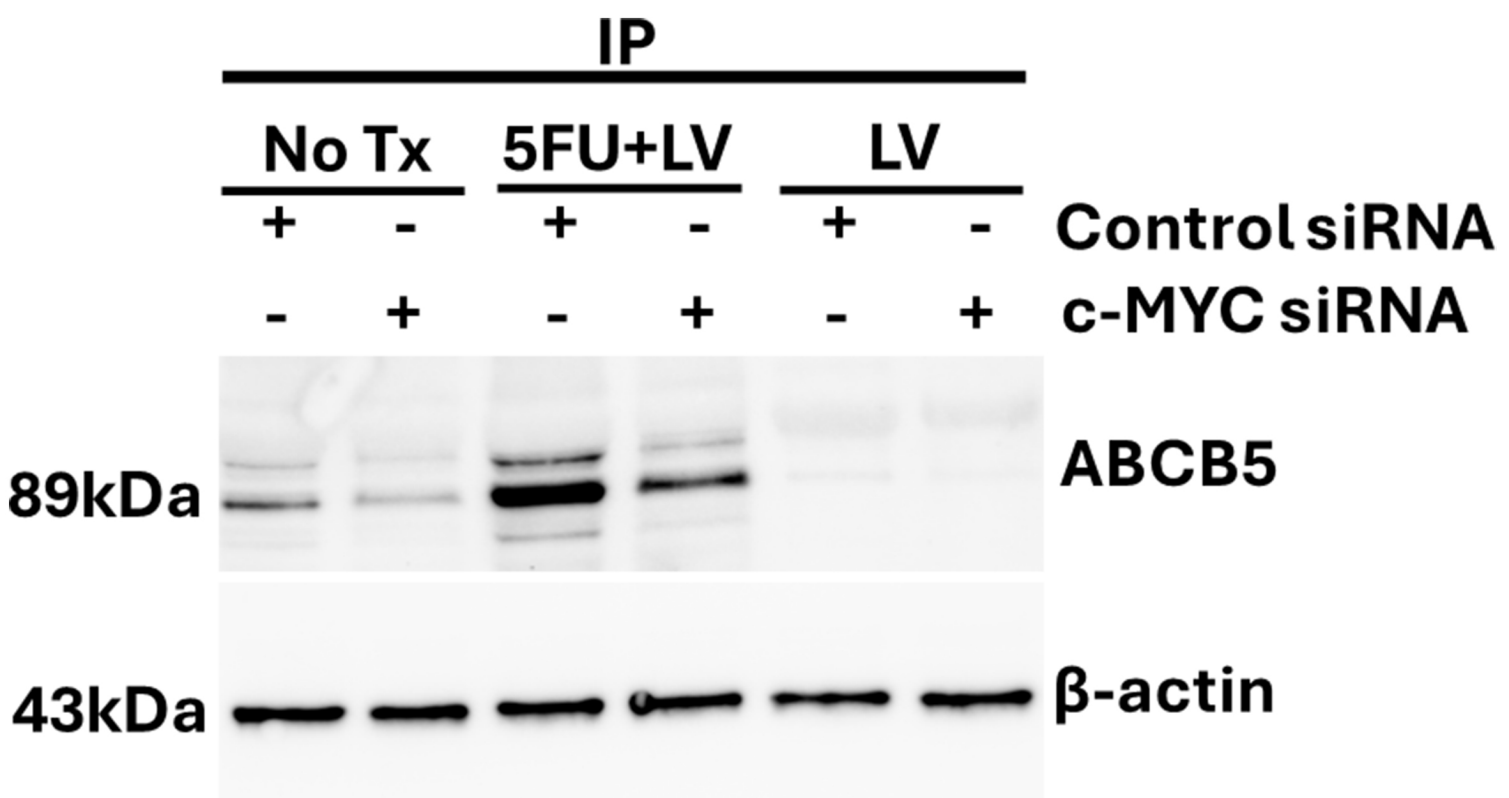
Immunoprecipitation of HCT116^R^ cell lysates reveals decreased ABCB5 following siRNA knockdown of Myc, consistent with increased ABCB5 in HCT116^R^ cells with 5-FU/LV treatment resulting from Myc-mediated transcription. Figure presentation software is the Bio-Rad ChemiDoc MP Imaging System (RRID: SCR_019037). 5-FU: 5-Fluorouracil; LV: leucovorin.

To demonstrate that ABCB5 is a Myc target in CRC^R^ cells, we performed siRNA-mediated knockdown of Myc followed by immunoprecipitation with anti-ABCB5. The proteins in the cell lysates were then separated by SDS-PAGE and probed to detect ABCB5 expression. As shown in Figure 5, treatment of HCT116^R^ cells with 5-FU/LV increased ABCB5 levels as expected; however, treatment with c-Myc siRNA reduced ABCB5 to basal levels comparable to untreated cells. These findings suggest that the increase in ABCB5 expression observed after 5-FU/LV treatment in HCT116^R^ cells is mediated by enhanced Myc-dependent transcription.

### CF10/LV induces Top1cc and DNA double-strand breaks in 5-FU/LV-resistant CRC cells

**Figure 6:**
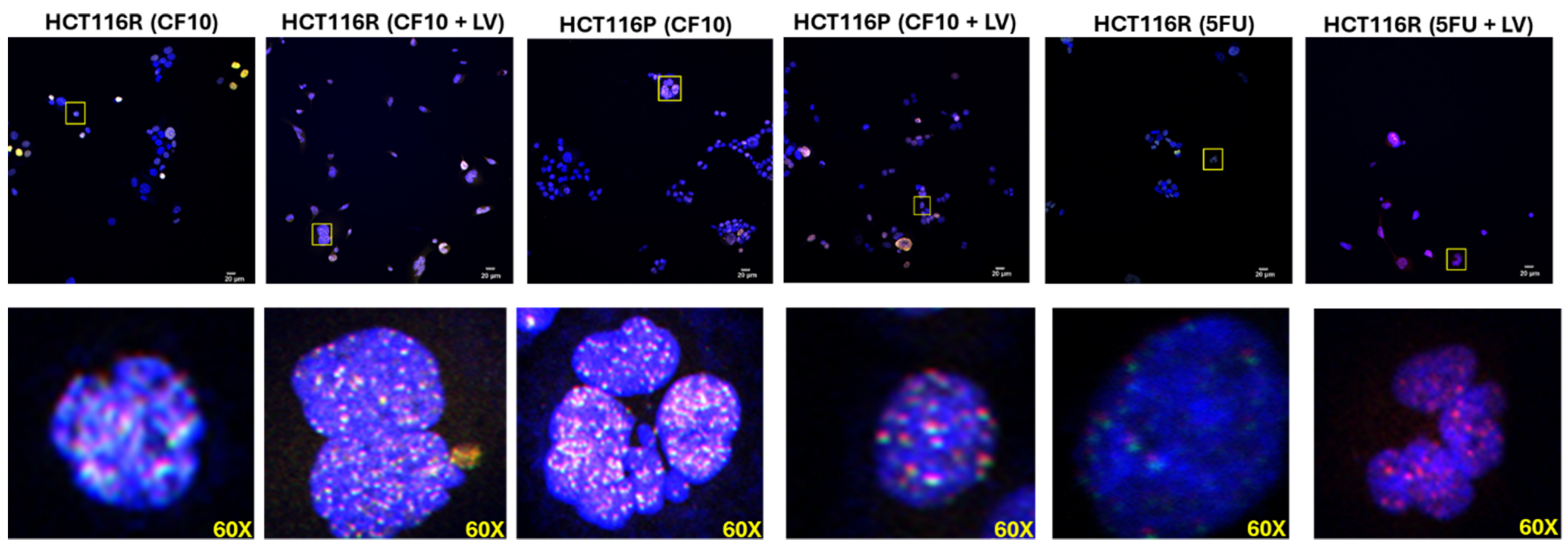
CF10/LV is effective at inducing Top1cc and γH2AX, a biomarker of DNA DSBs in both HCT116^R^ and HCT116^P^ cells, consistent with TS/Top1 dual targeting by CF10 in 5-FU/LV-resistant cells while 5-FU is relatively ineffective at causing DNA DSBs. Shown are merged images for DAPI (blue), γH2AX (green), and Top1cc (red) for the indicated cell lines and treatment (5-FU 10 mM, CF10 1 mM, LV 1 mM) for 24 h. Complete images are included in Supplementary Figures 11-16. Figure presentation software is Olympus Confocal Laser Scanning Microscope Fluoview FV3000 (RRID: SCR_017015). LV: Leucovorin; DSBs: double-strand breaks; TS: thymidylate synthase; 5-FU: 5-fluorouracil.

CF10 was shown to induce DNA topoisomerase 1 cleavage complex (Top1cc) formation in CRC cells^[21]^, and it displayed mechanistic similarities to Top1cc poisons such as camptothecin in a COMPARE analysis of data from the NCI60 cell line screen^[40,41]^. To determine if Top1cc formation and induction of DNA double-strand breaks (DSBs) also occur in CRC cells selected for 5-FU/LV-resistance upon treatment with CF10/LV, we performed immunofluorescence studies using antibodies specific for Top1cc^[38]^ and γH2AX, a biomarker of DNA DSBs [Figure 6, Supplementary Figures 11-16]. Top1cc were evident in nearly all HCT116^R^ cells treated with CF10/LV. Similarly, γH2AX immunostaining was evident in nearly all CF10/LV-treated HCT116-R cells, but not in those treated with 5-FU/LV. Our results are consistent with CF10/LV cytotoxicity being mediated through dual targeting of TS/Top1 in CRC cells selected for acquired 5-FU/LV resistance, whereas 5-FU/LV alone is not effective in causing DNA DSBs in these cells.

## DISCUSSION

This study characterizes three human CRC cell lines (HCT116, LS174T, HCT15) selected for acquired resistance to 5-FU/LV [Figure 1] in culture media that simulate human-like folate levels (FR media). All three CRC cell lines developed similar levels of 5-FU resistance (19.9-27.3-fold) [Figure 2 and Table 1], and these resistance levels were similar to those of CRC cell lines selected for resistance to 5-FU in standard media^[2]^. Because of its established role as a primary target of FP chemotherapy and mediator of 5-FU resistance, we evaluated TS expression at the protein and mRNA levels at baseline and in response to treatment with either 5-FU or a next-generation FP polymer CF10, with and without LV co-treatment. In contrast to previous studies, in which CRC cells selected for acquired resistance to 5-FU displayed elevated TS levels at baseline^[34]^, the CRC^R^ cells selected for acquired resistance to 5-FU/LV showed no significant increase in TS at either the mRNA or protein level in the absence of treatment. However, treatment with 5-FU/LV and, to a lesser extent, CF10/LV increased TS levels, and the effects were larger at the protein level than at the mRNA level [Figure 3, Supplementary Figures 1-5]. The mechanism by which 5-FU/LV treatment stimulates increased TS in CRC^R^ cells is under investigation in our laboratory. One possible explanation is treatment-induced stabilization of TS protein, preventing its degradation. TS can be stabilized against proteasome-mediated degradation through O-GlcNAc conjugation^[42]^, and it is possible that 5-FU/LV treatment enhances TS O-GlcNAc conjugation, thereby increasing protein stability.

In addition to its role in *de novo* thymidylate biosynthesis, TS is an RNA-binding protein that regulates its own expression and the expression of c-Myc and p53. Myc and p53 are key proteins that regulate cell proliferation and programmed cell death, and alterations in TS levels and in TS ternary complex formation could mediate drug resistance, in part by modulating c-Myc and p53 levels. In fact, c-Myc is significantly elevated in CRC^R^ cells selected for acquired resistance to 5-FU/LV relative to parental cells [Figure 4]. Although Myc levels were also altered in response to treatment, in contrast to the increased TS levels observed in response to 5-FU/LV treatment in CRC^R^ cells, both 5-FU/LV and CF10/LV significantly decreased Myc protein levels. Additionally, 5-FU/LV and CF10/LV treatments significantly decreased Myc mRNA levels, with Myc mRNA levels becoming barely detectable after CF10/LV treatment as measured by RT-qPCR. A possible explanation for these observations is that CF10/LV efficiently promotes the TS ternary complex, which relieves TS autoregulation^[27]^ and increases TS binding to Myc mRNA, thereby decreasing its translation. However, this process does not fully explain the observed decrease in Myc mRNA levels, particularly with CF10/LV treatment [Figure 4A-C, Supplementary Figures 7-10], indicating that additional processes are involved. Furthermore, while 5-FU/LV treatment reduces Myc levels in CRC^R^ cells, it simultaneously enhances Myc’s transcriptional activity with respect to ABCB5 expression [Figure 5]. Thus, both the levels and transcriptional activity of Myc are altered by 5-FU/LV treatment in CRC^R^ cells.

CF10 causes Top1cc and DNA DSB formation [Figure 6] and induces cell-cycle arrest, which could contribute to decreased c-Myc expression. In contrast, 5-FU/LV is less effective at promoting TS ternary complex formation [Figure 3] and induces lower levels of DNA DSBs [Figure 6], consistent with reduced cell-cycle arrest and correspondingly decreased Myc expression. While Myc regulates multiple targets involved in drug resistance, its direct transcriptional control of ABCB5 expression is known to modulate resistance to 5-FU in CRC cells^[30]^. In our study, we detected multiple higher-mobility bands for ABCB5 in Western blots of CRC^R^ cells [Figure 4D and E, Supplementary Figures 7-10], which may reflect alternative splicing events previously reported for ABCB5^[31]^. However, to our knowledge, these splice variants have not been implicated in acquired resistance to 5-FU/LV. Proteomic analyses are ongoing to identify the altered forms of ABCB5 in CRC^R^ cells. Interestingly, CF10/LV treatment decreased ABCB5 expression to barely detectable levels by RT-qPCR, and the putative alternatively spliced forms were undetectable following CF10/LV treatment. Collectively, these results suggest that CF10/LV downregulates the Myc/ABCB5 axis in CRC^R^ cells, thereby promoting strong cytotoxicity and overcoming acquired resistance to 5-FU/LV.

Acquired resistance to 5-FU/LV is a complex phenomenon, and our study necessarily focused on a limited number factors, particularly the induction of TS protein levels following 5-FU/LV treatment [Figure 3], and the increased basal levels of Myc in 5-FU/LV-resistant cells, which are modulated by FP treatment [Figure 4]. Myc has established roles in 5-FU resistance mediated through ABCB5, which we showed is differently affected by 5-FU/LV and CF10/LV treatments. Further investigation is needed to clarify how CF10/LV differentially regulates Myc levels relative to 5-FU/LV. In addition, Myc interacts with topoisomerase 1, and excessive Myc-topoisome activity can induce DNA damage and promote Myc degradation^[43]^. Top1 is also a target of CF10/LV [Figure 6], and future studies will investigate whether the decreased Myc levels observed with CF10/LV treatment involve this Myc-topoisome interaction. Another limitation of our study is that it was limited to cell models of 5-FU/LV resistance. Future research will extend these findings to *in vivo* models to validate the results and assess their potential impact on the standard of care for mCRC treatment.

The mortality associated with CRC results almost exclusively from metastatic disease. 5-FU/LV is widely used in regimens such as FOLFOX and FOLFIRI for the treatment of mCRC^[1]^, and the majority of patients initially respond to first-line chemotherapy. However, acquired resistance to 5-FU/LV almost invariably develops and long-term survival remains uncommon. In previous studies, we demonstrated that CF10 was very well tolerated *in vivo*, inducing less weight loss and causing fewer GI-tract and hematological toxicities compared to equivalent doses of 5-FU. Moreover, CF10 displayed superior antitumor activity relative to 5-FU in mouse models of primary CRC^[21]^ and in mouse and rat models of liver-metastatic CRC^[23]^. In these studies, we also evaluated whether CF10 remained effective in CRC cells selected for acquired resistance to 5-FU/LV. Remarkably, the resistance factors for CF10 were only 2.85- to 3.40-fold higher, approximately an order of magnitude lower than those for 5-FU in these cell lines [Table 1]. CF10 maintained its potency despite inducing similar increases in TS levels as 5-FU in CRC^R^ cells [Figure 3A and B, Supplementary Figures 7-10]. This retained efficacy was correlated with: (i) increased formation of the TS ternary complex in CRC^R^ cells [Figure 3A] and (ii) decreased TS enzymatic activity [Figure 3D]. The strong TS inhibition achieved by CF10/LV in CRC^R^ cells corresponded to the formation of Top1cc, as previously observed in CRC^[21]^ and pancreatic cancer cells^[22]^. This effect results from FdU misincorporation into DNA, which inhibits the religation step of Top1 catalysis^[40]^. Additionally, CF10/LV was significantly more potent than 5-FU/LV at inducing γH2AX foci [Figure 5], a biomarker of DNA DSBs, consistent with dual targeting of TS/Top1, causing replication stress and subsequent replication fork stalling and collapse. In summary, our results provide new insights into the mechanisms underlying acquired resistance to 5-FU/LV in CRC cells and indicate that CF10/LV is an effective alternative. Ongoing studies aim to test the efficacy of CF10/LV in *in vivo* models of acquired 5-FU/LV resistance and to advance CF10 into clinical trials. The activity demonstrated by CF10/LV in CRC cells selected for acquired resistance to 5-FU/LV supports its potential for clinical development, including use in patients previously treated with 5-FU/LV-based chemotherapy such as FOLFOX and FOLFIRI, potentially improving responses in third-line treatment of mCRC.

## DECLARATIONS

### Availability of data and materials

All datasets and materials generated in this study are available from the corresponding author upon reasonable request.

### Copyright

© The Author(s) 2025.
