# Supplementary material for: CF10/LV overcomes acquired resistance to 5-FU/LV in colorectal cancer cells through downregulation of the c-Myc/ABCB5 axis
Source: Cancer Drug Resist. 2025 Jul 15;8:35. doi: 10.20517/cdr.2025.76 (PMC12367397; doi:10.20517/cdr.2025.76)
Supplement: Supplementary file 1 [file cdr-8-35-SupplementaryMaterials.pdf]

Supplementary Materials

CF10/LV overcomes acquired resistance to 5-FU/LV in colorectal cancer cells through downregulation of the c-Myc/ABCB5 axis

Charles Chidi Okechukwu[1,2], William H. Gmeiner[2]

[1]Integrative Physiology and Pharmacology Graduate Program, Wake Forest University School of Medicine, Winston-Salem, NC 27157, USA.
[2]Department of Cancer Biology, Wake Forest University School of Medicine, Winston-Salem, NC 27157, USA.

Correspondence to: Prof. William H. Gmeiner, Department of Cancer Biology, Wake Forest University School of Medicine, Medical Center Blvd, Winston-Salem, NC 27157, USA. E-mail: bgmeiner@wakehealth.edu

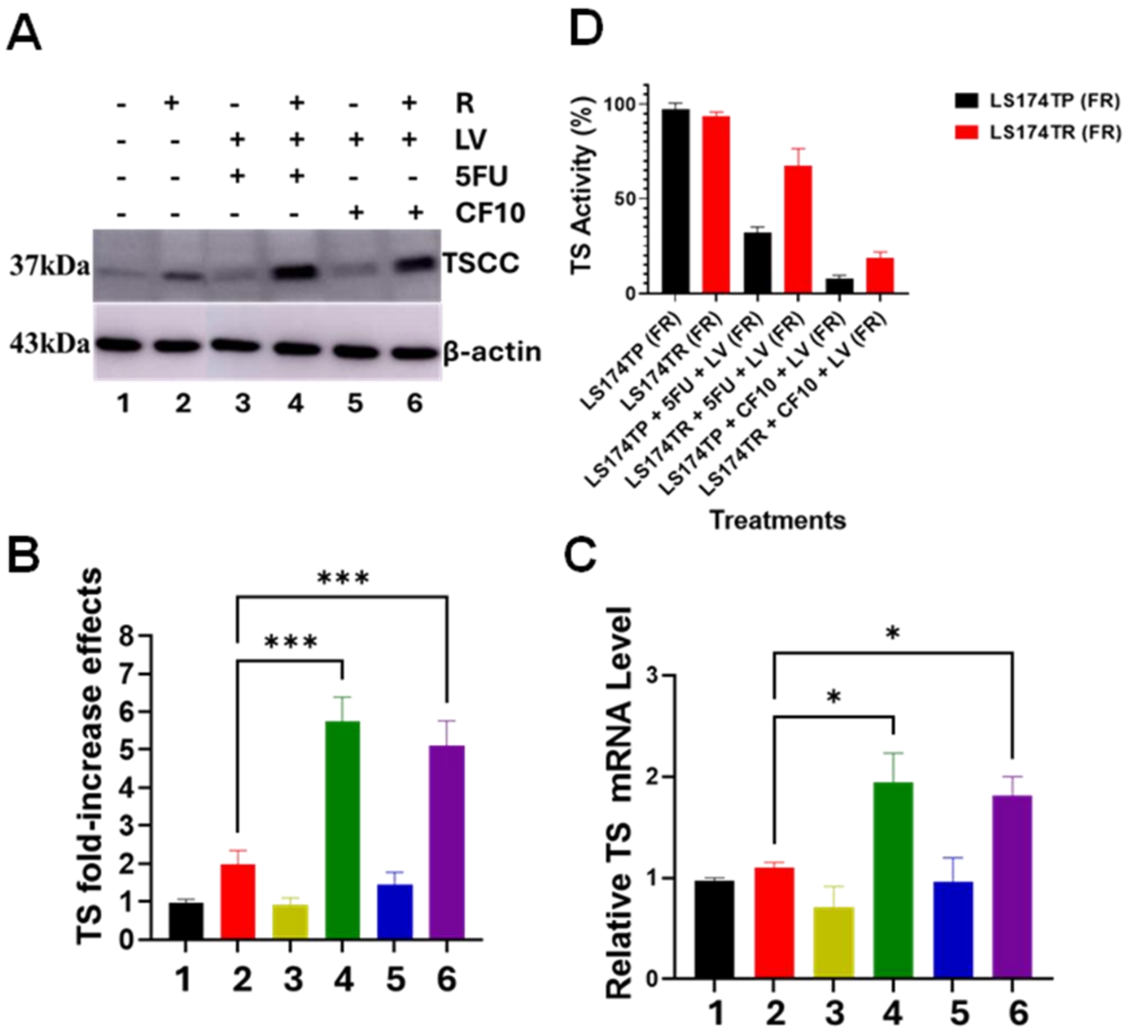

Supplementary Figure 1. 5-FU/LV (10 mM, 1 mM) and CF10/LV (1 mM, mM) treatment for 24h increases TS at both the protein (A and B) and mRNA (C) levels in $LS174T^R$ cells but only CF10/LV promotes ternary complex formation (TSCC) and efficient inhibition of TS enzymatic activity (D). $^{*}P < 0.05$; $^{***}P < 0.0001$.

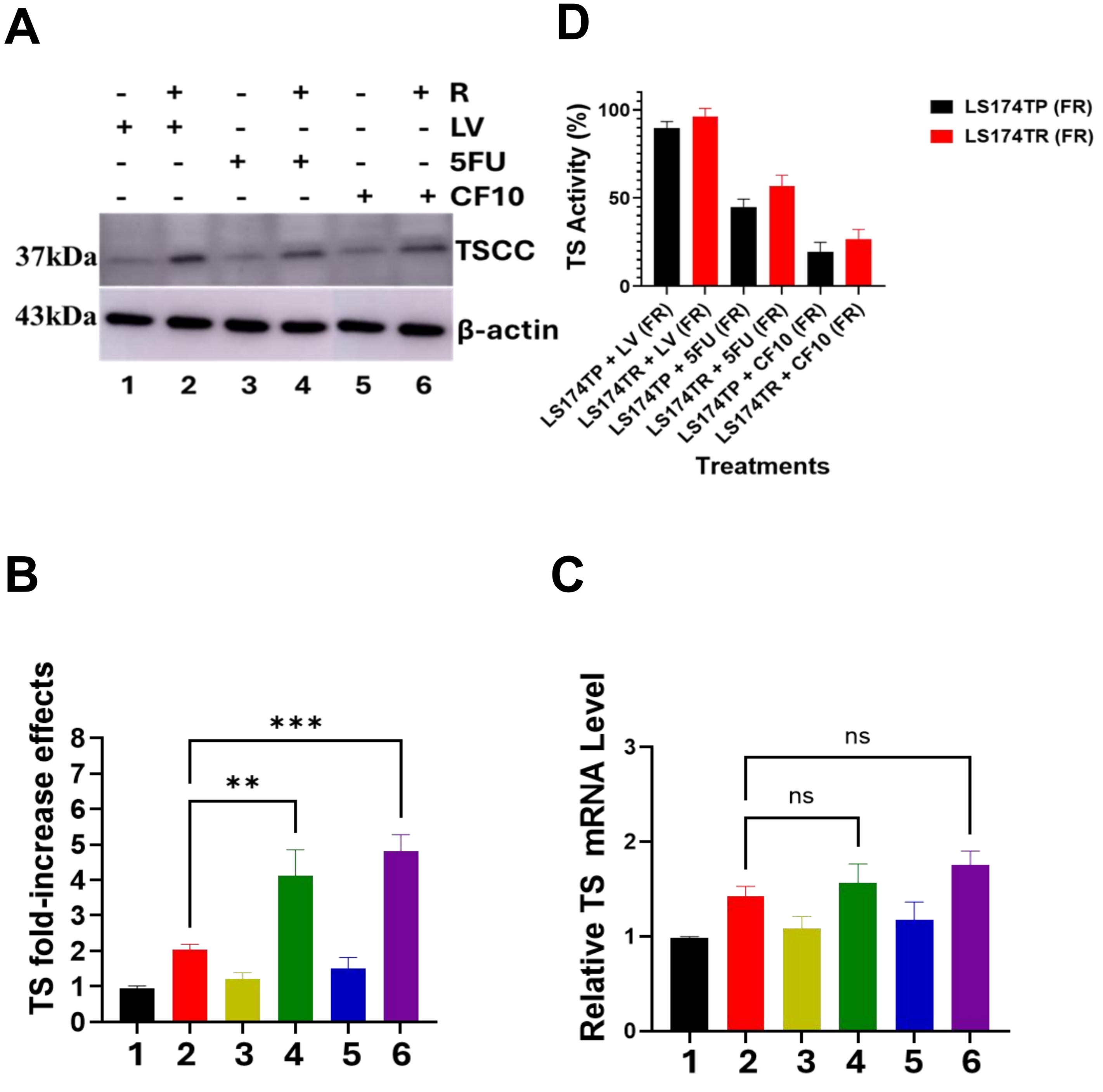

Supplementary Figure 2. 5-FU (10 mM) and CF10 (1 mM) treatment for 24 h increases TS at both the protein (A and B) and mRNA (C) levels in LS174T^R cells but only CF10 promotes ternary complex formation (TSCC) and efficient inhibition of TS enzymatic activity (D). $^{**}P<0.002$; $^{***}P < 0.0001$.

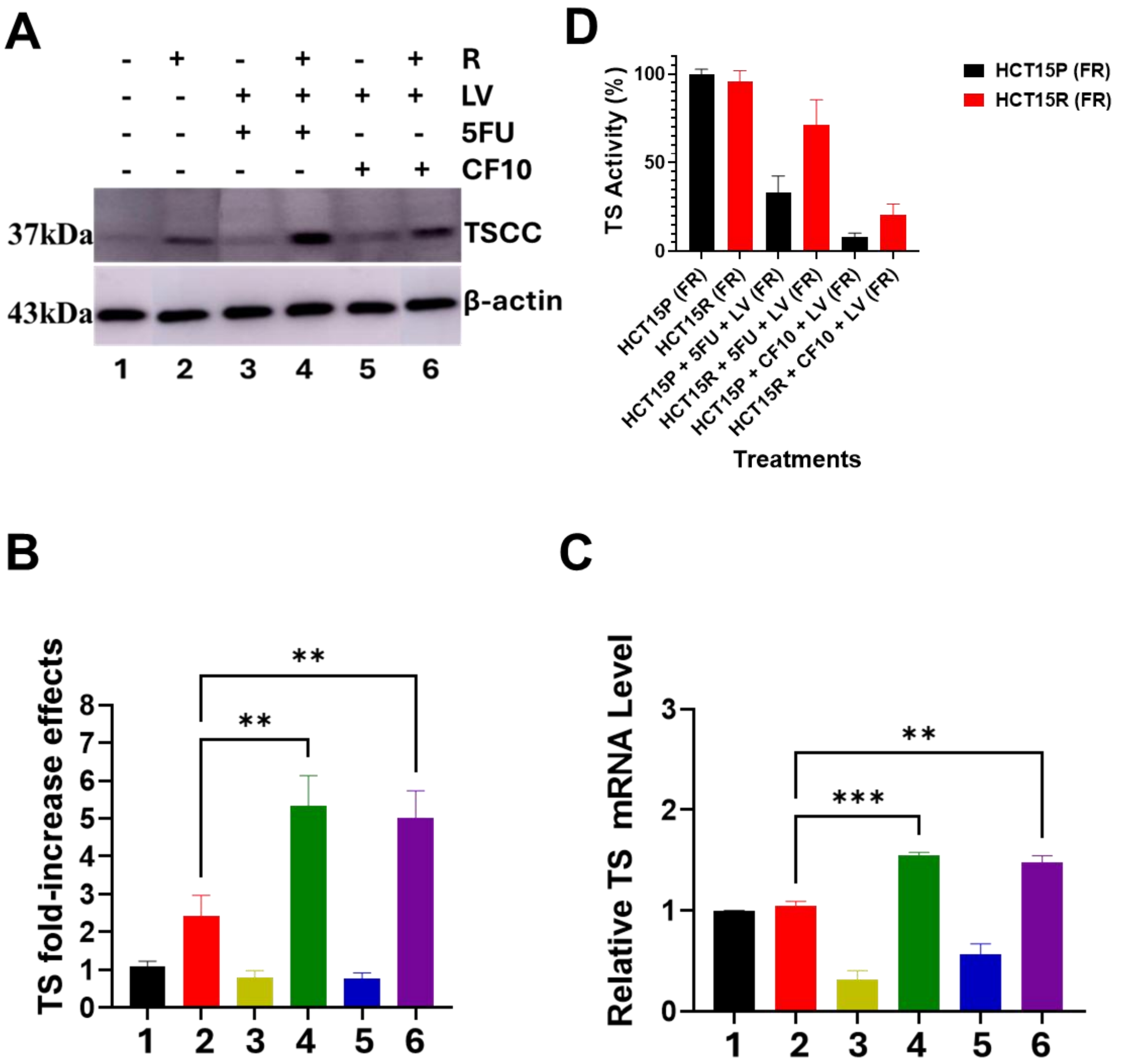

Supplementary Figure 3. 5-FU/LV (10 mM, 1 mM) and CF10/LV (1 mM, mM) treatment for 24h increases TS at both the protein (A and B) and mRNA (C) levels in HCT15^{R} cells but only CF10/LV promotes ternary complex formation (TSCC) and efficient inhibition of TS enzymatic activity (D). $^{**}P < 0.002$; $^{***}P < 0.0001$.

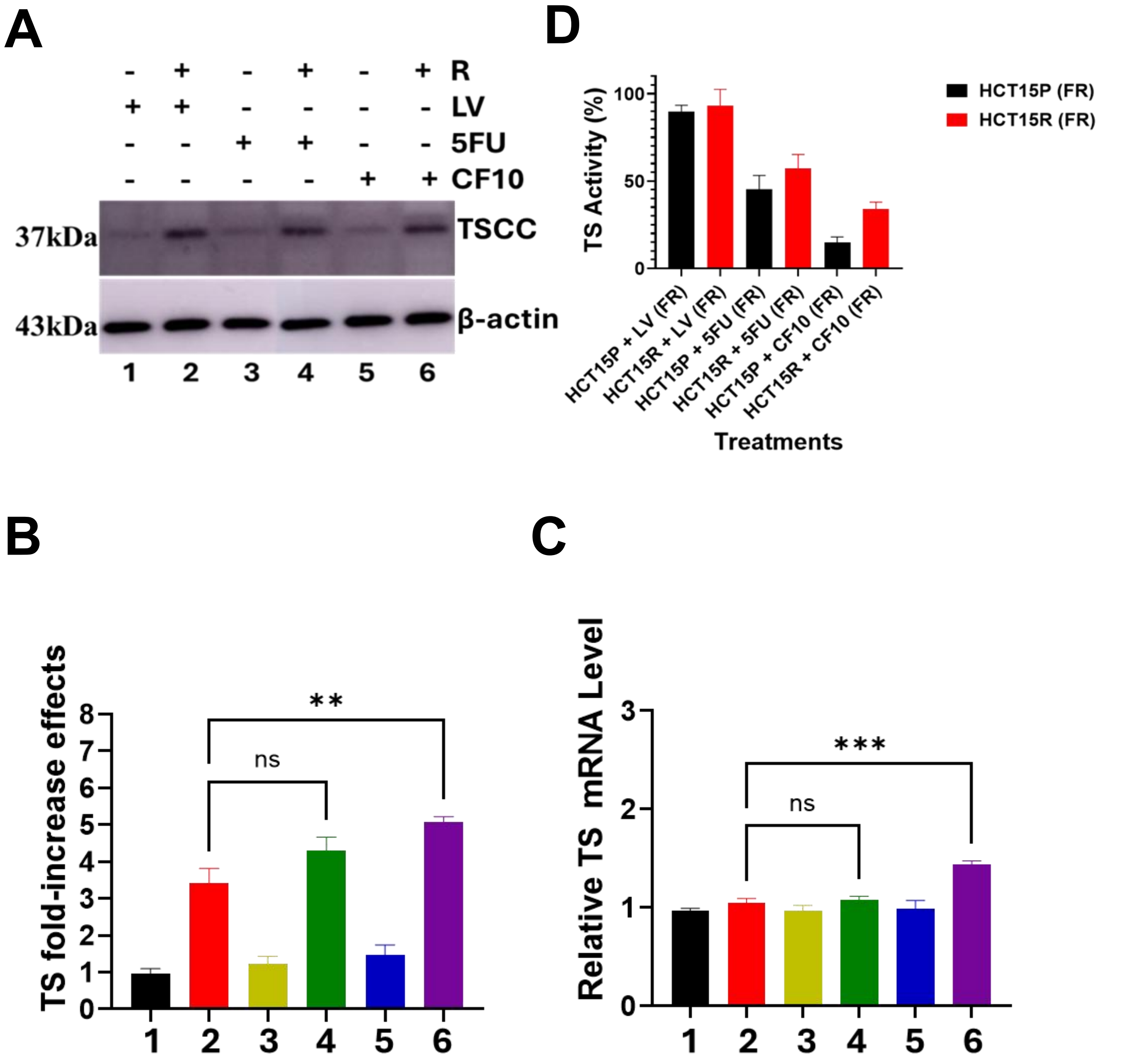

Supplementary Figure 4. 5-FU (10 mM) and CF10 (1 mM) treatment for 24h increases TS at both the protein (A and B) and mRNA (C) levels in $HCT15^R$ cells but only CF10 promotes ternary complex formation (TSCC) and efficient inhibition of TS enzymatic activity (D). $^{**}P < 0.002$; $^{***}P < 0.0001$.

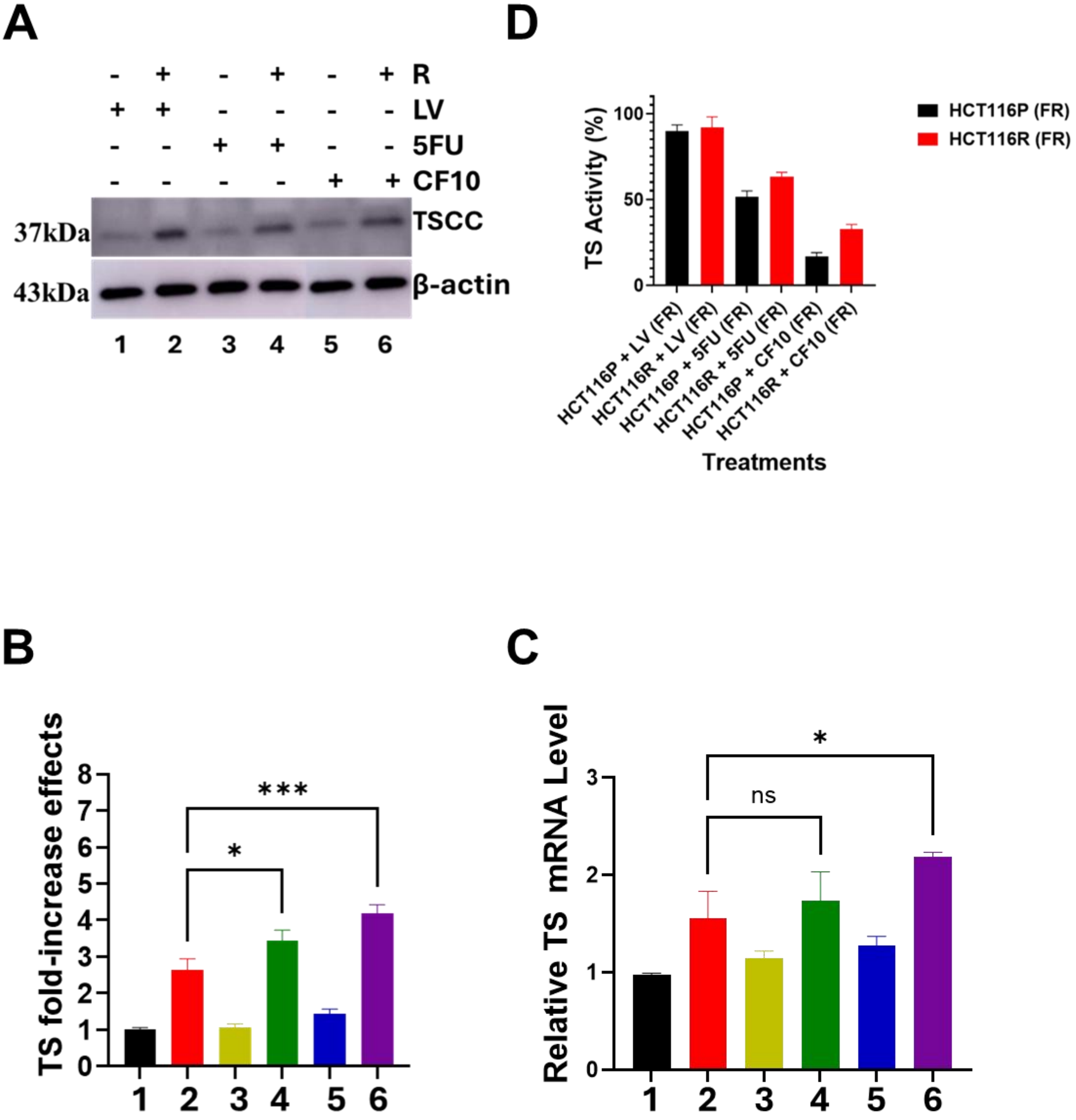

Supplementary Figure 5. 5-FU (10 mM) and CF10 (1 mM) treatment for 24h increases TS at both the protein (A and B) and mRNA (C) levels in HCT116^R cells but only CF10/LV promotes ternary complex formation (TSCC) and efficient inhibition of TS enzymatic activity (D). $^{*}P < 0.05$; $^{***}P < 0.0001$.

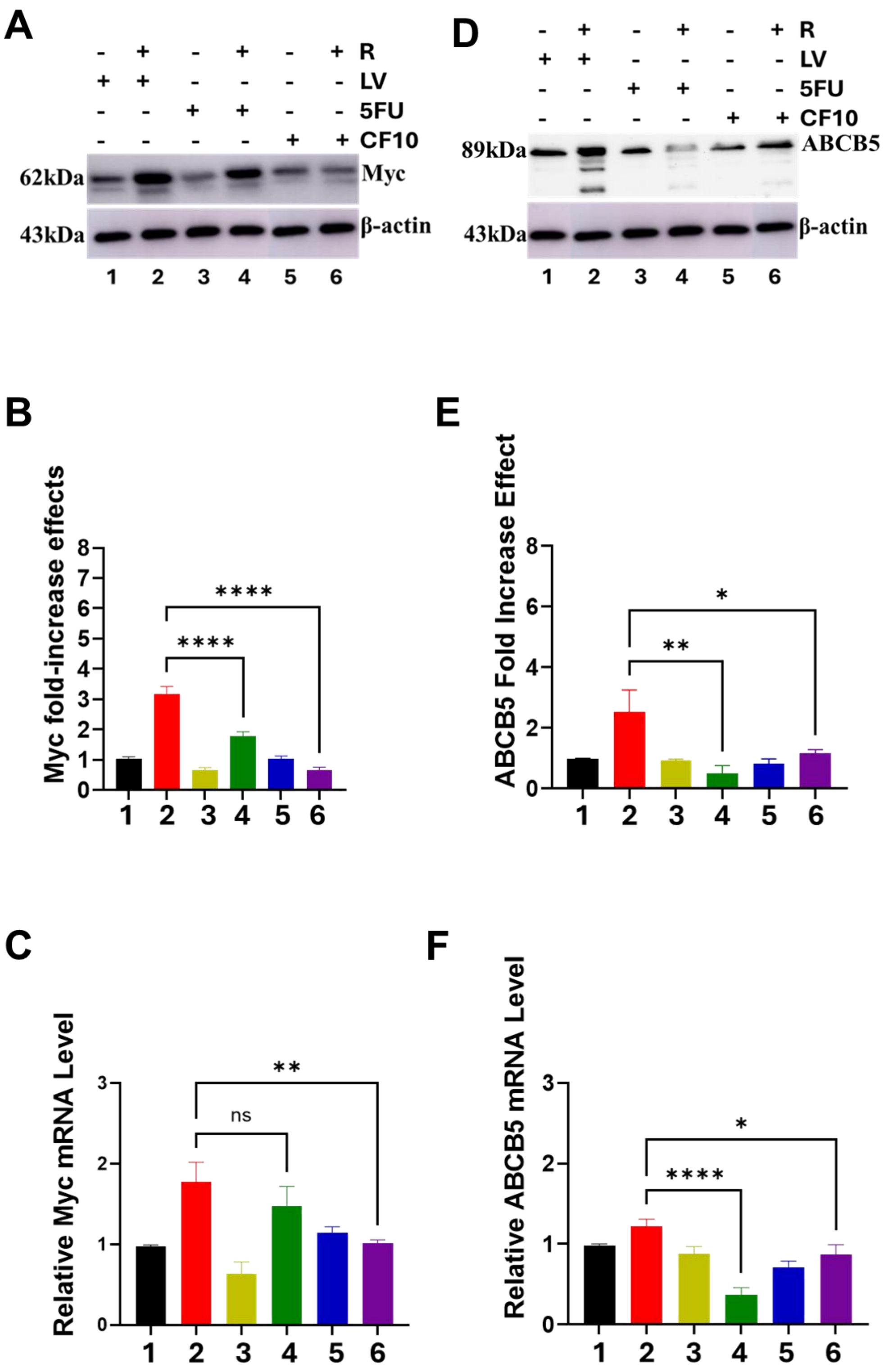

Supplementary Figure 6. Myc is elevated in 5-FU/LV-resistant HCT116^{R} cells, but 5-FU and CF10 decrease levels of Myc and the Myc-target ABCB5 at both the protein and mRNA level. Western blots for Myc (A) and ABCB5 (D). Quantification of the Western blot images by densitometry is shown for Myc (B) and ABCB5 (E). Quantification of mRNA levels by RT-qPCR is shown for Myc (C) and ABCB5 (F). $^{*}P < 0.03$, $^{**}P < 0.002$, $^{****}P < 0.0001$.

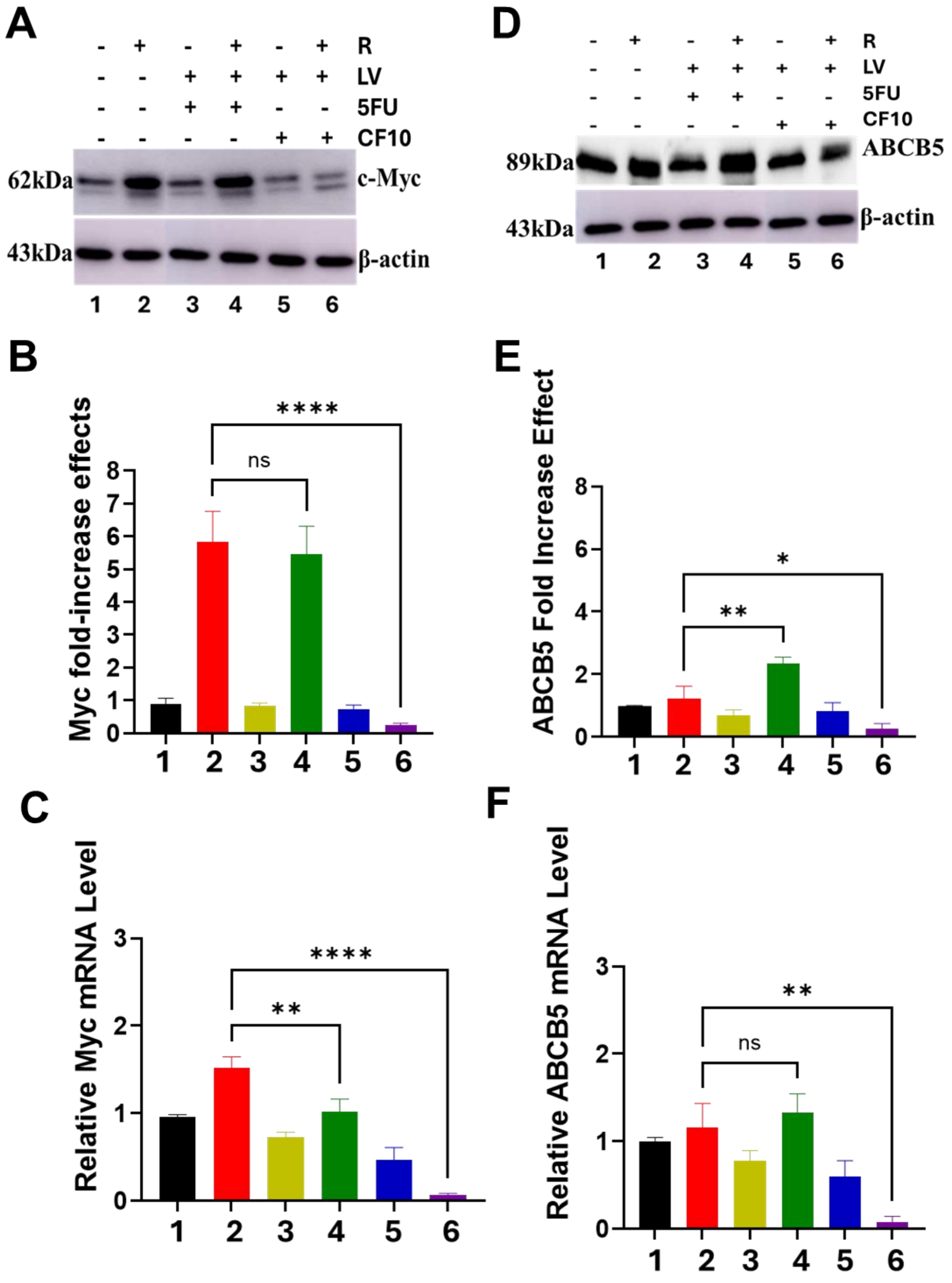

Supplementary Figure 7. Myc is elevated in 5-FU/LV-resistant LS174T^{R} cells, but CF10/LV decreases levels of Myc and the Myc-target ABCB5 at both the protein and mRNA level. Western blots for Myc (A) and ABCB5 (D). Quantification of the Western blot images by densitometry is shown for Myc (B) and ABCB5 (E). Quantification of mRNA levels by RT-qPCR is shown for Myc (C) and ABCB5 (F). $^{*}P < 0.03$, $^{**}P < 0.002$, $^{****}P < 0.0001$.

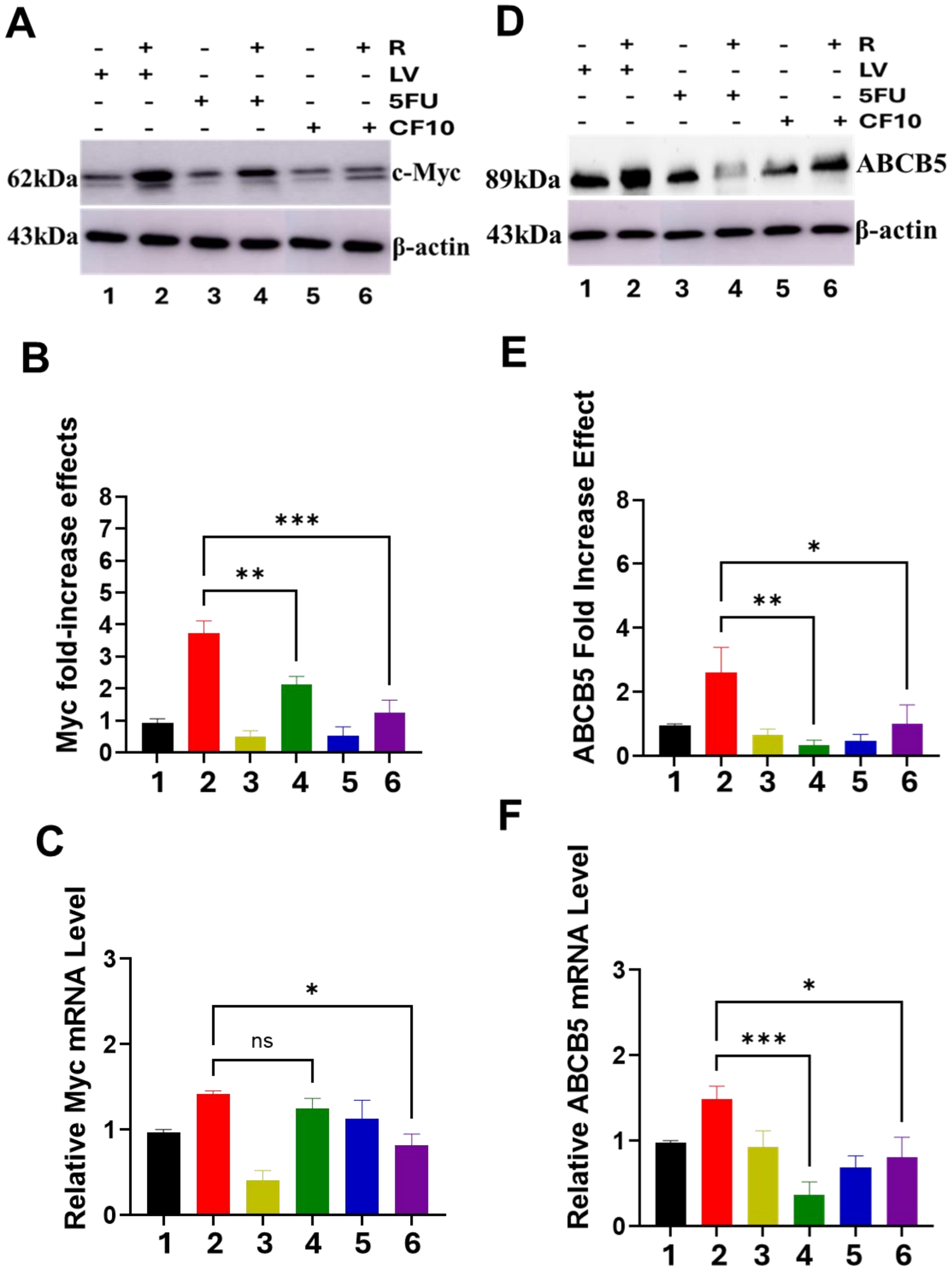

Supplementary Figure 8. Myc is elevated in 5-FU/LV-resistant LS174T^{R} cells, but 5-FU and CF10 decrease levels of Myc and the Myc-target ABCB5 at both the protein and mRNA level. Western blots for Myc (A) and ABCB5 (D). Quantification of the Western blot images by densitometry is shown for Myc (B) and ABCB5 (E). Quantification of mRNA levels by RT-qPCR is shown for Myc (C) and ABCB5 (F). $^{*}P < 0.03$, $^{**}P < 0.002$, $^{***}P < 0.002$.

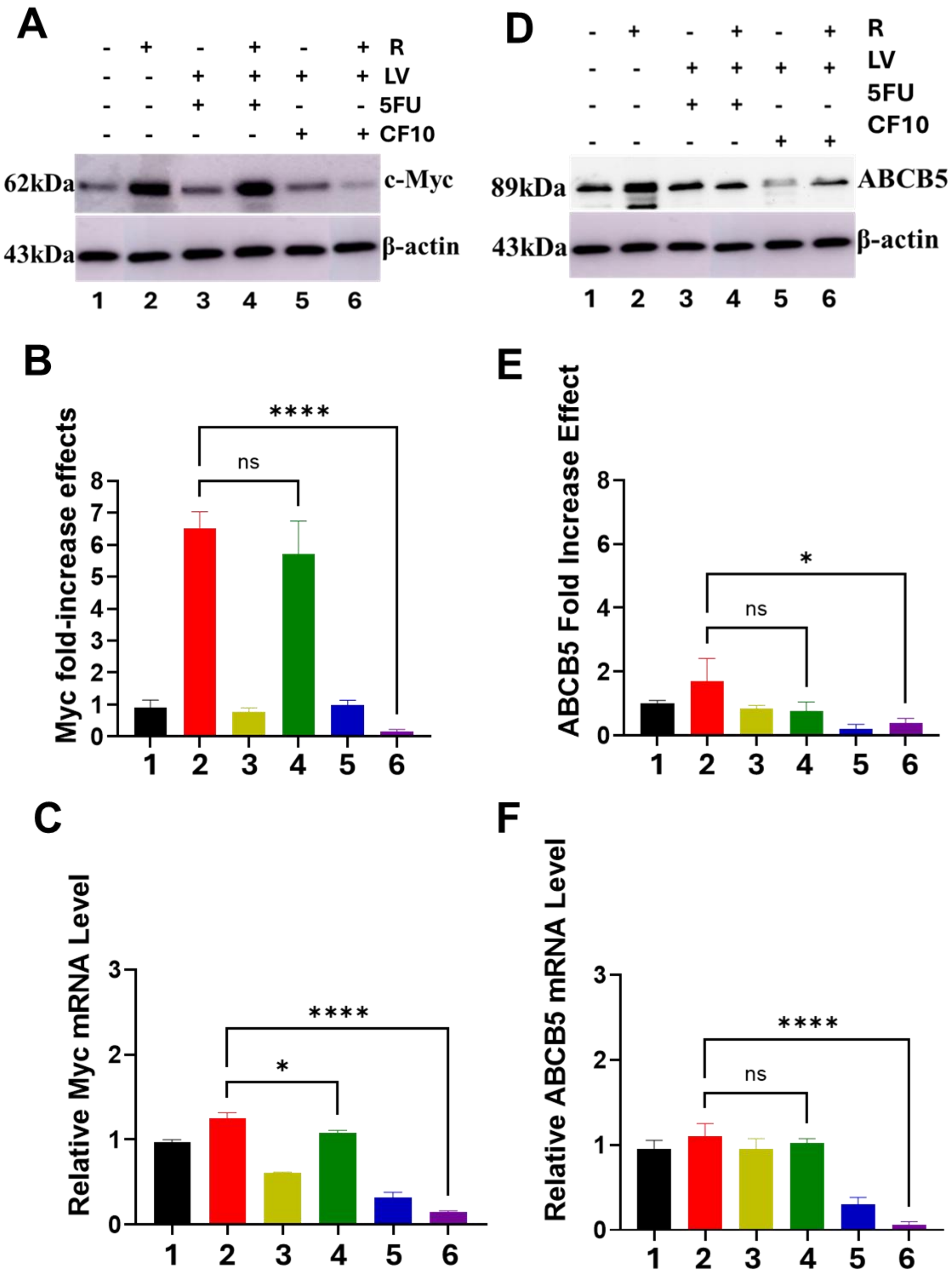

Supplementary Figure 9. Myc is elevated in 5-FU/LV-resistant HCT15^{R} cells, but CF10/LV decreases levels of Myc and the Myc-target ABCB5 at both the protein and mRNA level. Western blots for Myc (A) and ABCB5 (D). Quantification of the Western blot images by densitometry is shown for Myc (B) and ABCB5 (E). Quantification of mRNA levels by RT-qPCR is shown for Myc (C) and ABCB5 (F). $^{*}P < 0.03$, $^{****}P < 0.0001$.

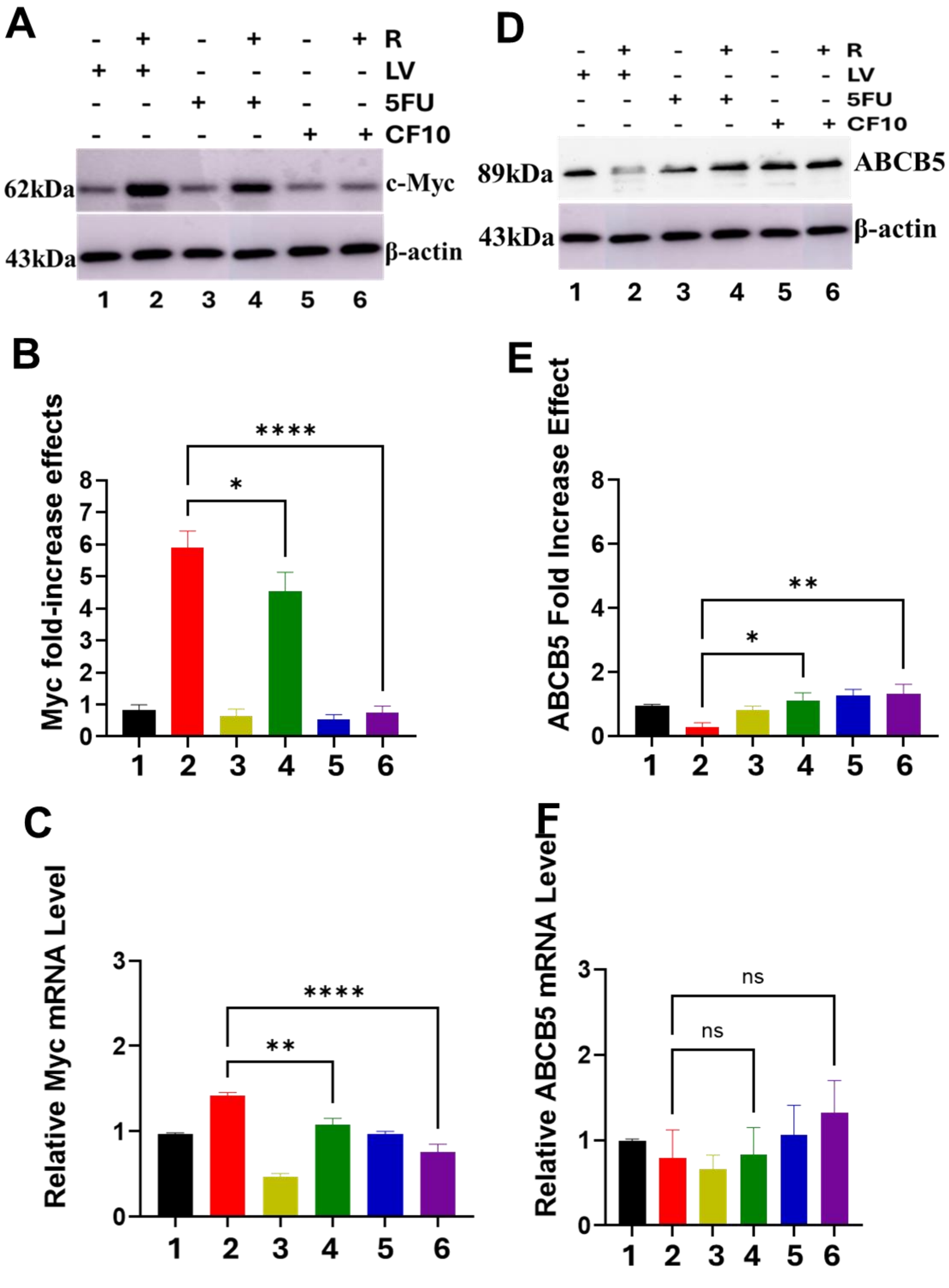

Supplementary Figure 10. Myc is elevated in 5-FU/LV-resistant HCT15^{R} cells, but 5-FU and CF10 decrease levels of Myc and the Myc-target ABCB5 at both the protein and mRNA level. Western blots for Myc (A) and ABCB5 (D). Quantification of the Western blot images by densitometry is shown for Myc (B) and ABCB5 (E). Quantification of mRNA levels by RT-qPCR is shown for Myc (C) and ABCB5 (F). $^{*}P < 0.03$, $^{**}P < 0.002$, $^{****}P < 0.0001$.

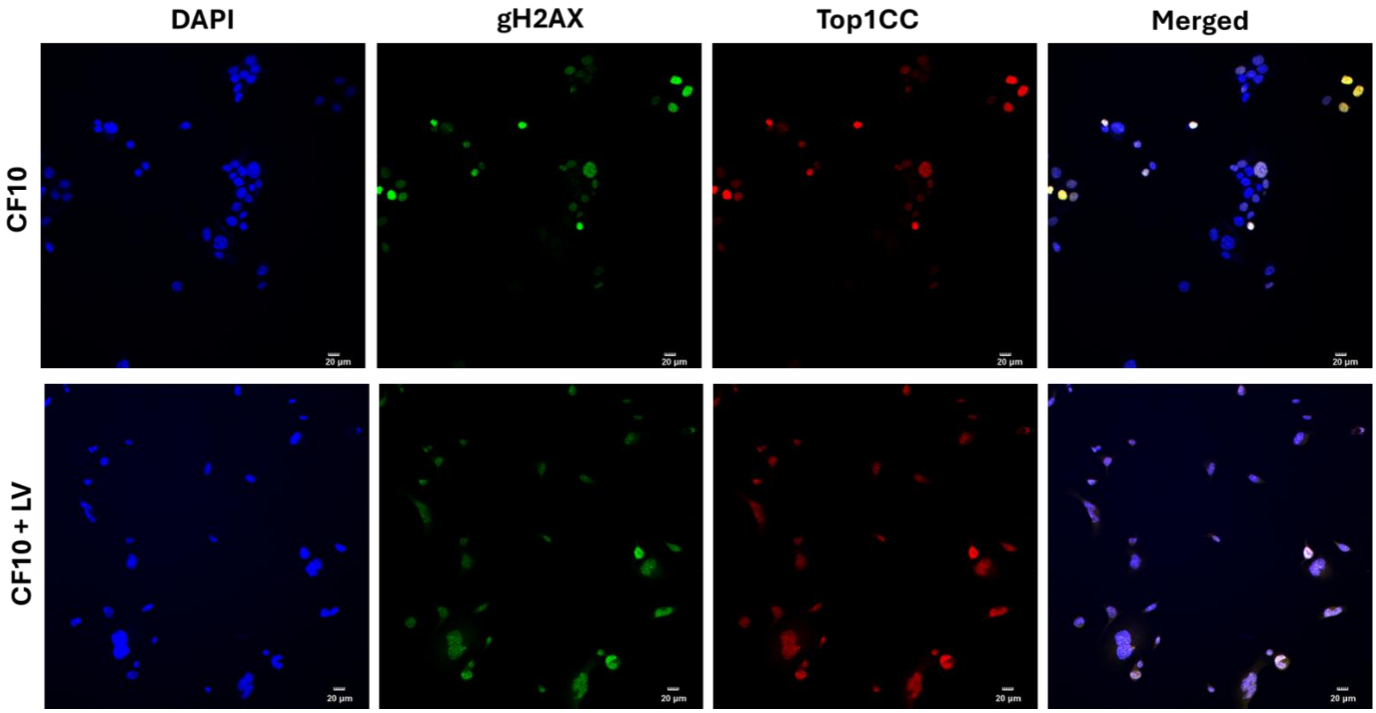

Supplementary Figure 11. Immunofluorescence Imaging of pH2AX and Top1cc in HCT116R cells following treatment with CF10 or CF10 + LV.

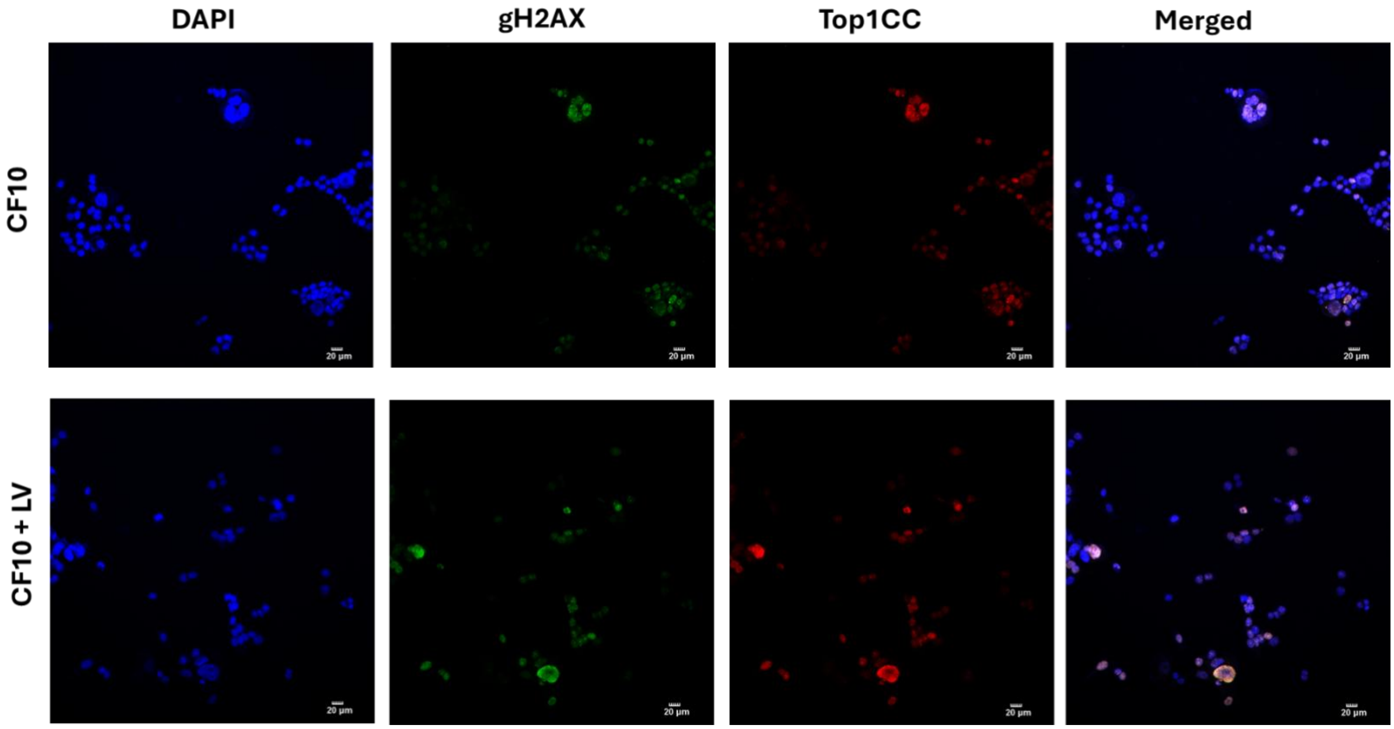

Supplementary Figure 12. Immunofluorescence Imaging of pH2AX and Top1cc in HCT116P cells following treatment with CF10 or CF10 + LV.

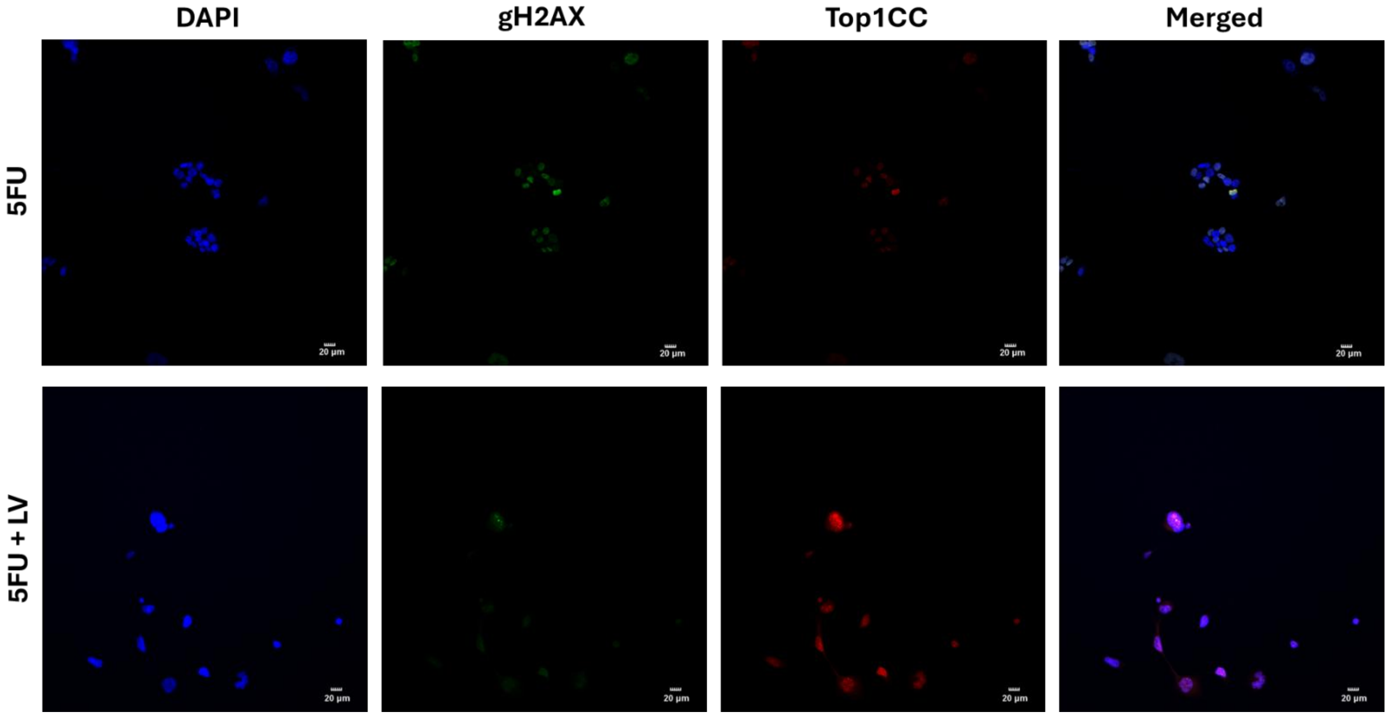

Supplementary Figure 13. Immunofluorescence Imaging of pH2AX and Top1cc in HCT116R cells following treatment with 5FU or 5FU + LV.

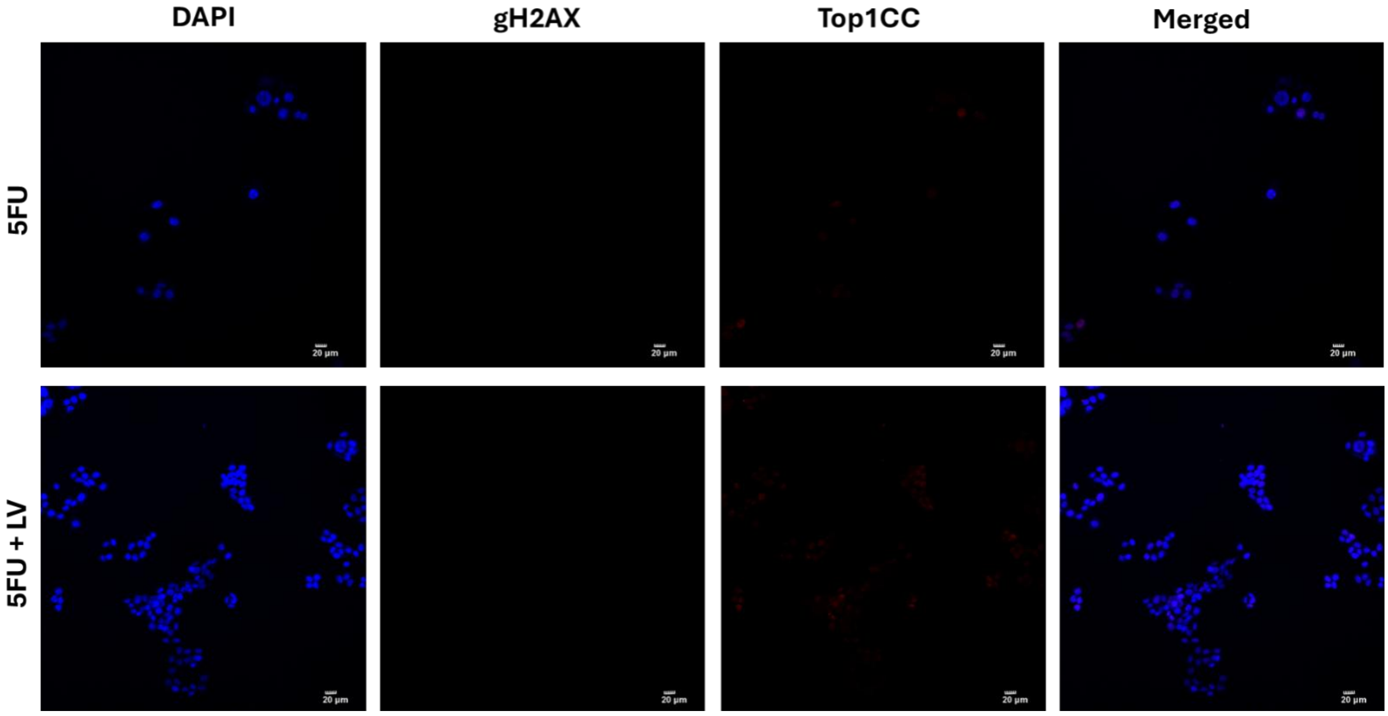

Supplementary Figure 14. Immunofluorescence Imaging of pH2AX and Top1cc in HCT116P cells following treatment with 5FU or 5FU + LV.

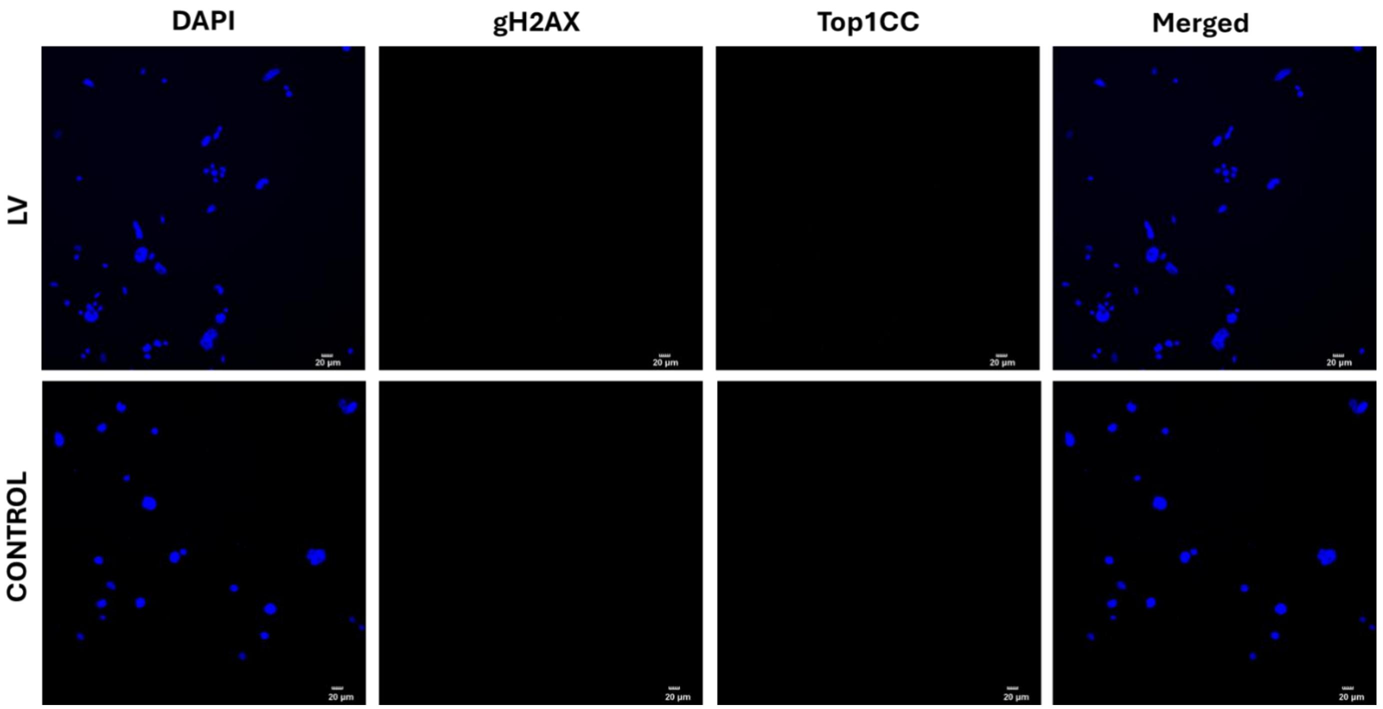

Supplementary Figure 15. Immunofluorescence Imaging of pH2AX and Top1cc in HCT116R cells following treatment with LV or no Treatment.

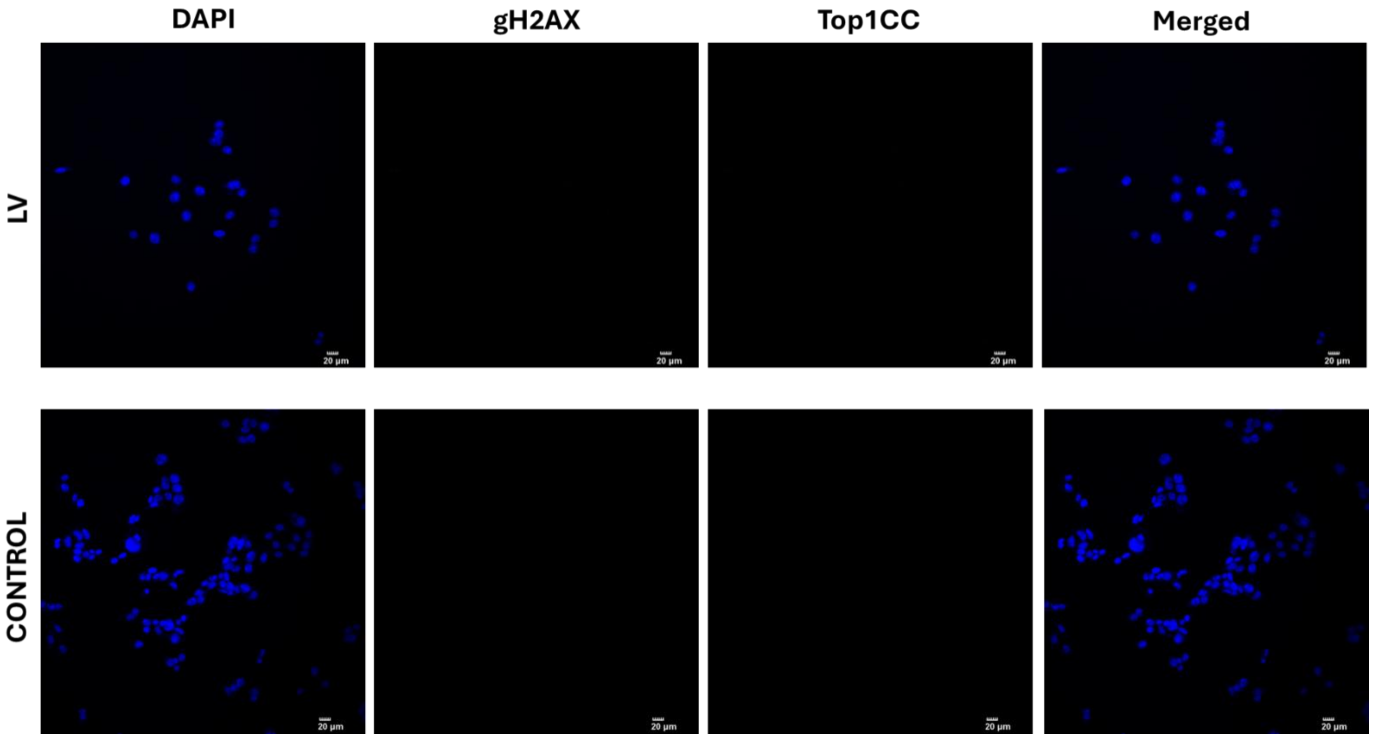

Supplementary Figure 16. Immunofluorescence Imaging of pH2AX and Top1cc in HCT116P cells following treatment with LV or no treatment.
